# Modulating Efficiency and Color of Thermally Activated Delayed Fluorescence by Rationalizing the Substitution Effect

**DOI:** 10.1021/acs.jctc.4c00009

**Published:** 2024-05-13

**Authors:** Alejandro Jodra, Marco Marazzi, Luis Manuel Frutos, Cristina García-Iriepa

**Affiliations:** † Departamento de Química Analítica, Química Física e Ingeniería Química, Grupo de Reactividad y Estructura Molecular (RESMOL), Universidad de Alcalá, Ctra. Madrid-Barcelona, Km 33.600, Alcalá de Henares, Madrid 28871, Spain; ‡ Instituto de Investigación Química “Andrés M. del Río” (IQAR), Universidad de Alcalá, Ctra. Madrid-Barcelona, Km 33.600, Alcalá de Henares, Madrid 28871, Spain

## Abstract

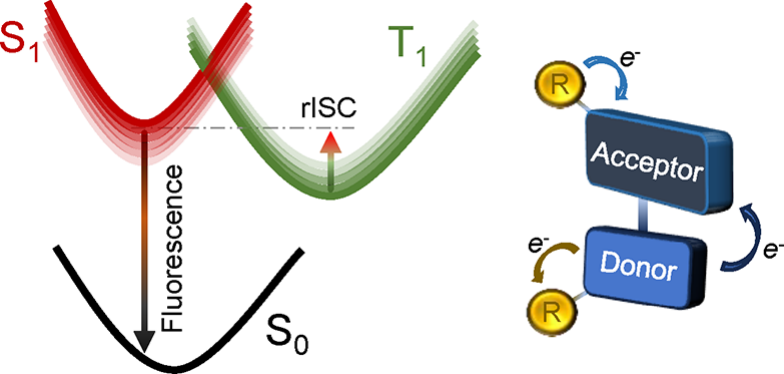

Thermally activated delayed fluorescence (TADF) constitutes the process by which third-generation organic light-emitting diodes (OLEDs) are being designed and produced. Despite several years of trial-and-error attempts, mainly driven by chemical intuition about how to improve a certain aspect of the process, few studies focused on the in-depth description of its two key properties: efficiency of the T_1_ → S_1_ intersystem crossing and further S_1_ → S_0_ emission. Here, by means of a newly developed theoretical formalism, we propose a systematic rationalization of the substituent effect in a paradigmatic class of OLED compounds, based on phenothiazine-dibenzothiophene-*S*,*S*-dioxide, known as PTZ-DBTO2. Our methodology allows to discern among geometrical and electronic effects induced by the substituent, deeply understanding the relationships existing between charge transfer, spin density, geometrical deformations, and energy modulations between electronic states. By our results, we can finally elucidate, depending on the substituent, the fate of the overall TADF process, quantitatively assessing its efficiency and predicting the color emission. Moreover, the general terms by which this methodology was developed allow its application to any chromophore of interest.

## 1 Introduction

Thermally activated delayed fluorescence (TADF) is a process that, in recent years, has seen a great deal of development due to its varied and numerous applications in the area of organic electronics.^1−4^ In particular, the TADF process has been considered to develop novel families of organic light-emitting diodes (OLEDs) with important advantages compared to inorganic LEDs.^5−9^ Indeed, OLEDs usually show higher efficiency, lower power consumption, and flexible and ultrathin display characteristics, moreover resulting in an economically cheaper solution, since expensive metals are not required (e.g., platinum, iridium).^10^

**Scheme 1:**
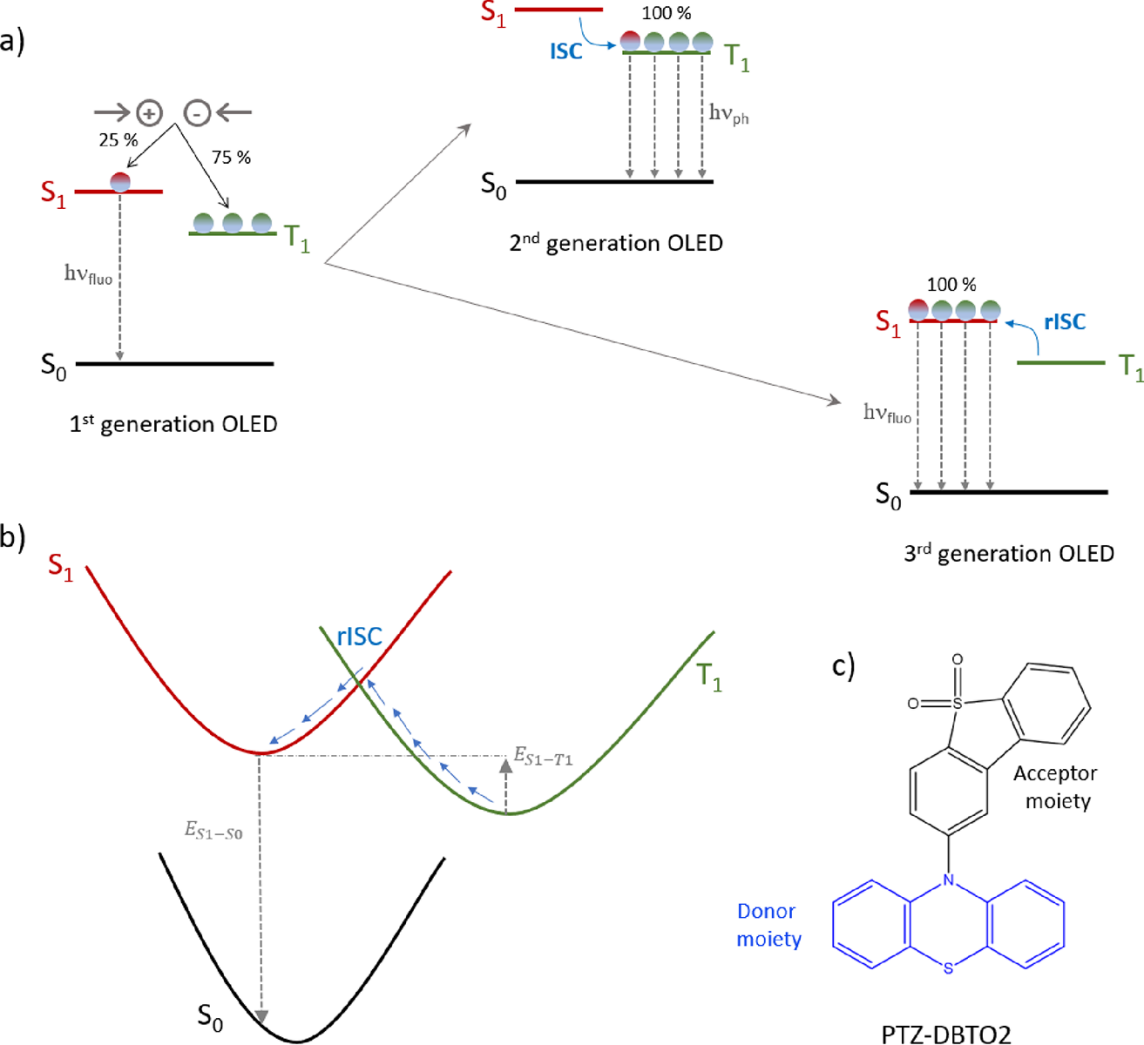
(a) Jablonski Diagram-Based Scheme of the Three OLED Generations: Hole and Electron Injection Followed by Fluorescence (First Generation), ISC, and Phosphorescence (Second Generation), rISC, and Fluorescence (Third Generation); (b) Simplified One-Dimensional Potential Energy Surfaces Showing the TADF Process, Including the T_1_ → S_1_ rISC and Fluorescence Emission Paths (Solid Blue and Dotted Gray Arrows)—the S_1_–T_1_ (*E*_S1–T1_) and S_1_–S_0_ (*E*_S1–S0_) Energy Differences to Be Modulated Are also Shown; (c) Structure of the PTZ-DBTO2 System under Study, Depicting in Blue the Electron Donor Moiety and in Black the Electron Acceptor Moiety

In OLEDs, the electroluminescent layer is composed of organic semiconductors, typically small-size organic molecules. OLED-based screens can be found nowadays in smartphones and TVs, mainly due to a low consumption of electric energy, high brightness, and color contrast, as well as a low device weight, compared to traditional screens.^11^ The working mechanism of an OLED is as follows: a voltage is applied to the organic semiconductor, resulting in the injection of electrons and holes that flow across the semiconductor until combining to form a highly localized exciton. Since both electrons and holes are Fermions, there are four different possibilities to combine their spin: three combinations (75%) create a triplet electronic state and only one combination (25%) creates a singlet electronic state, usually corresponding to T_1_ and S_1_, respectively, i.e., the lowest energy excited states, with S_0_ referred to as the ground state. Since first-generation OLEDs were based on fluorescence (Scheme 1a), this means that their efficiency is severely limited to a maximum of 25%. A possible solution is the increase of the spin–orbit coupling (SOC) allowing the intersystem crossing (ISC) from the singlet to the triplet manifold, in this case from S_1_ to T_1_. This was feasible thanks to the introduction of heavy metals as iridium.^12^ In this way, second-generation OLEDs could, in principle, reach a 100% efficiency based on phosphorescence (Scheme 1a). Nevertheless, phosphorescence has two drawbacks: it is usually much less intense than fluorescence, and the emissive transition corresponds to lower energy. This is because, in most of the known conjugated systems, T_1_ is lower in energy than S_1_ due to electron exchange interactions, as stated by the Hund’s rule.^13^ Moreover, apart from phosphorescence, nonradiative decay from T_1_ to S_0_ could also happen.^14^ To overcome these limitations, TADF processes were invoked as a conceptual way to increase OLED fluorescence (third-generation OLEDs). Indeed, the TADF process consists in taking advantage of thermal energy to allow T_1_ → S_1_ reverse intersystem crossing (rISC), reestablishing a consistent fluorescence emission (Scheme 1a,b).^14−16^ The rISC mechanism is not yet fully understood, but it is often described in terms of a kinetic model drawn on the basis of Marcus theory,^17−19^ initially proposed to explain the rate of an electron transfer reaction.^20^ In any case, in the point of view of OLED efficiency, it appears clear that increasing T_1_ → S_1_ rISC could, depending on the molecular system, induce a fluorescent enhancement, overcoming the original 25% barrier.

By analyzing the process, we can discern two key variables that regulate the overall efficiency. One is the S_1_–T_1_ SOC, which is related to the nature of the states involved in the process.^21−23^ It is known that the electronic nature of these states, whether they are charge transfer (CT) or locally excited (LE) states, can regulate this coupling, the optimal condition requiring two states of different electronic nature, where possibly the transition involves a change in orbital orientation, as stated by El-Sayed.^24,25^ The second variable determining the T_1_ → S_1_ nonradiative efficiency is the energy difference between these states, with T_1_ and S_1_ energy degeneracy constituting the most promising condition. Both variables were already found to be optimal and explain the phenomenon of delayed fluorescence in paradigmatic organic molecules in solution as benzophenone.^26,27^ For the specific case of the rISC process in TADF-related compounds, S_1_–T_1_ energy differences of 4.6 kcal/mol (0.20 eV) or lower have been found to be small enough to ensure a considerable rISC efficiency.^14,21,28−31^

Since it would be highly desirable to predict the TADF efficiency, many computational studies have been dedicated to rationalizing and deeply analyzing the TADF process.^32−34^ Among the two aforementioned variables ruling such efficiency (S_1_–T_1_ SOC and energy difference values), most of the studies focused on how to reach S_1_–T_1_ energy degeneration.^35,36^ For this aim, diverse properties of TADF derivatives have been examined to explain their effect.^37^ On the one hand, the role of the electronic nature of S_1_ and T_1_^38^ has been explored by proposing the two-state model,^39^ by which it was concluded that the S_1_–T_1_ energy difference depends solely on the exchange integral between the HOMO and LUMO orbitals. The higher the CT character described by these orbitals, the smaller the value of the integral and hence the smaller the S_1_–T_1_ energy difference. However, the above model does not consider the option of having LE states while numerous experimental studies show systems in which T_1_ is an LE state.^40,41^ Therefore, more complex models have been constructed including such states (four-state model).^39^ On the other hand, structural modifications have been proposed to influence the S_1_–T_1_ energy degeneracy.^42−46^ In particular, the dihedral angle formed between donor and acceptor moieties has been studied and related to the rISC process efficiency.^47−49^ Hence, with the goal of achieving S_1_–T_1_ energy degeneracy by modulating the electronic nature of the states involved, different systematic experiments were attempted introducing substituents to modify the aforementioned dihedral angle in chromophores originating TADF.^31,46,50−53^

Apart from enhancing the rISC efficiency, another crucial point in designing OLEDs is the emission color.^54,55^ Since all colors can be obtained as an appropriate combination of red, green, and blue (RGB) emitters, the aim is to obtain molecules capable of shining, each one, in the red, green, or blue ranges, possibly through a sharp and intense emission. In other words, the technological goal is to obtain proper RGB OLED emitters. However, the vast majority of OLED emitters based on the TADF principle emit red light^56−59^ while some emit green light and very few examples of blue emitters do exist.^60−63^

All in all, although many experimental and some limited theoretical efforts were spent to improve the design of TADF-based OLED emitters, this field clearly lacks some general theoretical predictive tool to describe the effect of substitution patterns on the process efficiency and the emitted color, given a certain chromophoric building block. Hence, this work tries to fill in this gap, presenting a theoretical method to investigate, at the same time, the possible increase of the rISC efficiency and the emission color modulation.

In order to prove the reliability of the obtained results, such method was applied to study a paradigmatic class of compounds showing TADF properties, based on the phenothiazine-dibenzothiophene-S,S-dioxide derivative (PTZ-DBTO2, Scheme 1c).^14,50,64,65^ Substituents of diverse electronic nature (−NH_2_, −NO_2_, −CH_3_) introduced in a wide variety of PTZ-DBTO2 positions have been computationally studied to show the match with the proposed theoretical method. The novelty of this study is that the substitution effect is theoretically rationalized in a way that both the electronic nature of S_1_ and T_1_ states, together with the geometrical change due to substitutions, are considered at the same time. The results show that both factors can be studied independently, although they are strongly related, and both clearly influence the rISC efficiency and color emission modulation. Moreover, this study proposes novel PTZ-DBTO2 derivatives with significantly enhanced rISC efficiency while explaining the physical limits behind the difficulties in obtaining blue emission. The generality of the proposed method, which can in principle be applied to any potential emitter in combination with any possible chemical substituent, should hopefully help in the design of significantly improved TADF-based OLED emitters.

## 2 Methods

### 2.1 Electronic Structure methods

The geometries of the PTZ-DBTO2 reference compound and of its 33 derivatives under study (see Figure S3 in Supporting Information) have been optimized in the electronic ground state (S_0_) and lowest triplet state (T_1_) at the density functional theory (DFT) level, while the singlet excited state (S_1_) was calculated by time-dependent (TD)-DFT. Moreover, T_1_ was also calculated by TD-DFT to check the effect on the S_1_–T_1_ gap (see Figure S13). Guided by a previous computational work^14^ on the class of compounds under study and by a benchmark of functionals and basis sets (see Tables S1 and S2, respectively, in the Supporting Information), the M06-2X^66^ functional and the SVP basis set have been selected.

**Figure 1:**
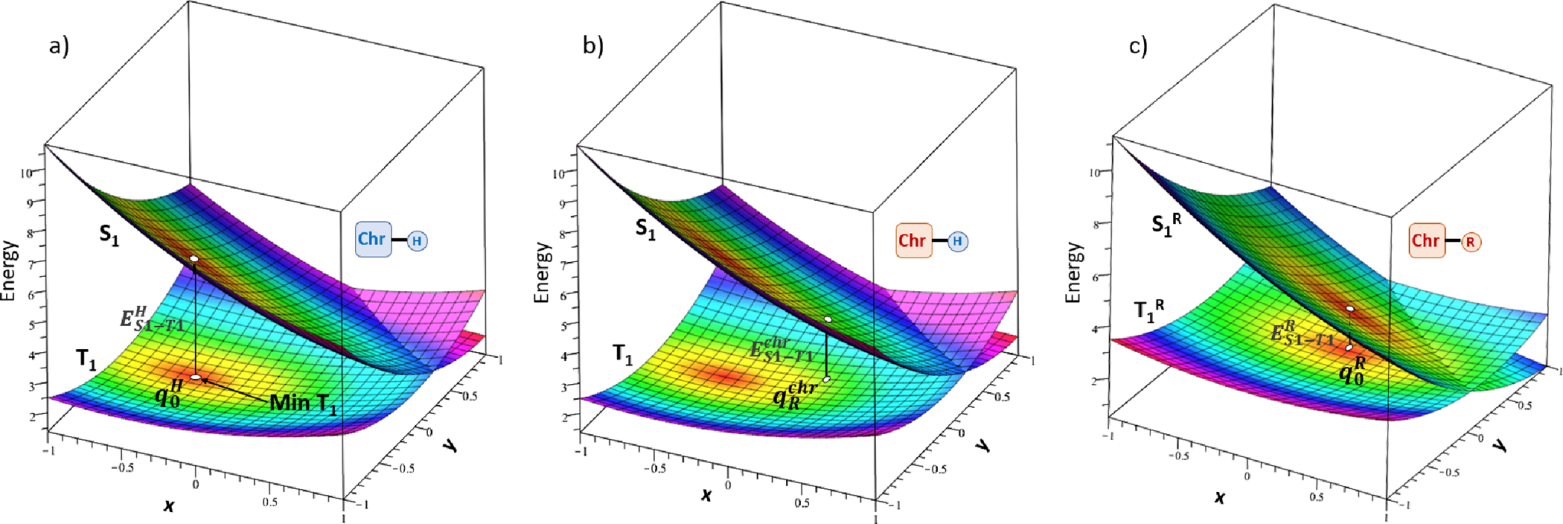
T_1_ and S_1_ schematic two-dimensional PESs (a, b) of the unsubstituted derivative (Chr-H) as a function of two internal coordinates (**x** and **y**), showing details for (a) the T_1_ minimum and (b) the T_1_ geometry corresponding to the coordinates of the chromophore altered by substitution, i.e., the point corresponding to the prediction of the T_1_ minimum displacement due to substitution. (c) T_1_ and S_1_ PESs of the substituted derivative (Chr-R), proving the T_1_ minimum displacement due to substitution. In all cases, the corresponding S_1_–T_1_ energy difference is depicted. The color code corresponds to the T_1_ energetics (red: energy minimum), and it is maintained for the S_1_ surface.

Moreover, these calculations have been performed in the gas phase since the solvent effects are sensitive to the type of solvent and PTZ-DBTO2 conformation (see Table S3 in the Supporting Information). Especially, implicit toluene and dichloromethane are considered through linear response (integral equation formalism variant of the polarizable continuum model, IEFPCM,^67^ as reported in similar studies)^14^ and by corrected linear response^68^ methods. These calculations have been performed with the Gaussian16^69^ suite of programs. SOC values have been computed at the same level of theory using the ORCA 4.2.1 package.^70^ A quantitative characterization of the CT character has been performed by CT indexes, as calculated through the TheoDORE package,^71^ by considering the molecule divided into two parts, the phenothiazine and the dibenzothiophene-S,S-dioxide moieties (Figure 1c).

Regarding the calculation of the spin density index, the spin density on each atom has been extracted from the T_1_ calculations. To compute such index, the molecule has been divided in the same two moieties considered for CT, phenothiazine, and dibenzothiophene-S,S-dioxide. Then, the spin density of each moiety, calculated as the sum of the spin densities on each atom, has been computed. Finally, the values have been normalized in order that the sum of the normalized spin density index calculated for the two moieties is 1. Hence, a spin density index of 1 for the phenothiazine (donor) moiety means that the spin density is completely located in this part whereas a value of 0.5 indicates that the spin density is equally distributed among the two moieties.

### 2.2 Theoretical Model of the Substituent Effect

In this work, the substituent effect is first considered for the variation of the energy difference between S_1_ and T_1_ potential energy surfaces. To do this, we first split each molecular system into two parts and consequently also the internal coordinates of the system, the chromophore (with coordinates ***q***^chr^) and the substituent (with coordinates ***q***^subst^). When the substituent is the hydrogen atom H, we consider this system as unsubstituted and it corresponds to our reference. This reference system is characterized by a minimum energy geometry in the potential energy surface (PES) of T_1_, with coordinates ***q***_0_^H^, and a corresponding singlet–triplet energy difference *E*_S1–T1_^H^ (Figure 1a). When H is replaced by a substituent group, the PES changes accordingly and so the minimum energy geometry changes to a new geometry with coordinates ***q***_0_^R^ together with the corresponding singlet–triplet energy difference *E*_S1 – T1_^R^ (Figure 1c). This work is focused on understanding the nature of these changes depending on the introduced substituent. It should be remarked that the methodology presented hereafter is going to be detailed considering the S_1_–T_1_ energy difference, but it could be applied to any other energy difference. Indeed, a simpler formulation was already proposed by some of the authors to modulate the S_0_–S_1_ excitation energy (i.e., the electronic absorption spectrum) of *S*-nitrosothiols, where the concept of “structural substituent effect” was introduced^72^ and later extended in a more general formalism including the electronic effects and their relation with internal forces.^73^ In graphic terms, this methodology explains how to approximate T_1_ minimum energy coordinates (and the corresponding S_1_–T_1_ energy difference) of a substituted chromophore (Chr-R, Figure 1c) by using the PESs built for the unsubstituted chromophore (Chr-H, Figure 1a) and separating the coordinates of chromophore and substituent moieties (Figure 1b).

In the following, we present the formalism to rationalize the effect of substitution on the S_1_–T_1_ energy gap. More specifically, the variation of this energy gap, that can be directly related to the activation energy required to populate the S_1_ state within the Marcus theory (see Figure S12), is decomposed into two terms, the first one due to the structural effect induced by the substituent (i.e., the variation of the minimum energy structure, as explained above) and the second one due to the different global stabilization of S_1_ and T_1_ states induced by substitution.

The energy terms are labeled with superscript R or H, indicating if the energy is referred to the substituted or unsubstituted system, respectively, while the electronic state is denoted with a subscript. Additionally, in parentheses, the geometry for which the energy is determined is indicated. For instance, the energy in the S_1_ state for a substituted system (R) determined at its T_1_ minimum energy structure results in the following notation: *E*_T1_^R^(***q***_0_^R^).

With the aim of understanding the substituent effect on the S_1_–T_1_ energy gap (i.e., the vertical energy from T_1_ minimum to S_1_), we define its variation due to substitution as
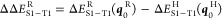
1where
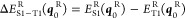
2
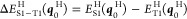
3

It has to be remarked that, while ***q***_0_^R^ refers to the T_1_ minimum energy structure for the substituted system, ***q***_0_^H^ refers to the equivalent point for the unsubstituted system. Hence, we have two different systems characterized by their respective energy minima, the unsubstituted original chromophore (Chr-H, Figure 1a) and the substituted (Chr-R, Figure 1c) one.

When a chromophore is chemically substituted, the excitation energy of the new system can be understood as the excitation energy of the unsubstituted system plus an additional perturbation energy induced by the substituent. Such perturbation energy can be expanded in terms of a function series up to the first order, centered at the Chr-H equilibrium geometry (i.e., ***q***_0_^H^ ≡ **0**):
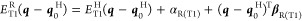
4where α_R(T1)_ can be interpreted as the constant shift (independent of the coordinates) that the presence of the -R substituent provokes on the T_1_ state of the unsubstituted (Chr-H) system. Additionally, the **β**_R(T1)_ term provides the first variation of the energy as a function of the coordinates. Its physical interpretation becomes clear if we determine the equilibrium position of the Chr-R system. Indeed, by calculating the gradient of the previous equation, we obtain
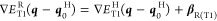
5When *q* = *q*_0_^R^, this gradient equals zero, and therefore,
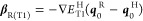
6where ***q***_0_^R^ – ***q***_0_^H^ is the vector providing the structural displacement of the system after substitution. Hence, **β**_R(T1)_ is the force induced by the substituent, as it has been defined elsewhere,^73^ responsible for the change in the structure upon substitution, from ***q***_0_^H^ to ***q***_0_^R^.

Within these assumptions, the energies of the T_1_ and S_1_ states can be evaluated at the T_1_ equilibrium geometry of both the substituted system (***q***_0_^R^) and the unsubstituted system (***q***_0_^H^):
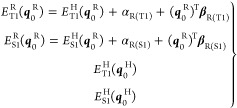
7

Therefore, the S_1_–T_1_ energy gap for ***q***_0_^R^ and ***q***_0_^H^ is
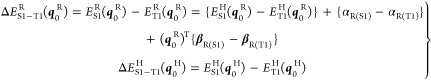
8

Concerning the substituted system, it can be therefore concluded that the S_1_–T_1_ energy gap is expressed as a function containing three terms: a first term depending only on the energy of the unsubstituted system, plus two additional terms corresponding to the zeroth- and first-order effect of the substituent to the energy of each state.

Finally, the variation of the S_1_–T_1_ energy gap due to substitution, initially defined by eq 1, can be expressed as
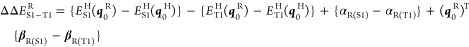
9

The α_R(S1)_ – α_R(T1)_ term provides the differential stabilization of the two states due to the presence of the substituent, while the term (***q***_0_^R^)^T^{**β**_R(S1)_ – **β**_R(T1)_} provides the change of the differential structural effect within the chromophore due to the force induced by the substituent. In the following, we limit our expansion in the energy difference to the zeroth-order term, hence assuming that the substituent-induced forces are essentially the same in both S_1_ and T_1_ states, and therefore, **β**_R(S1)_ – **β**_R(T1)_ ≈ 0. This assumption comes from the facts that the substituent is not involved in the excitation (both in S_1_ and T_1_ states) and that the chromophore is similarly affected in both states, resulting in similar substituent-induced forces in both states.

Concluding, the variation of the S_1_–T_1_ energy gap expressed by eq 9 can be simplified as follows:
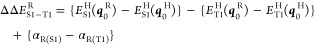
10where the first two terms correspond to the geometrical effect that the substitution causes to the chromophore while the third term corresponds to the zeroth-order differential effect of the substituent on the electronic energy of both S_1_ and T_1_ states. For simplicity, this expression can be written as
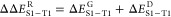
11with Δ*E*_S1–T1_^G^ = {*E*_S1_^H^(***q***_0_^R^) – *E*_S1_^H^(***q***_0_^H^)} – {*E*_T1_^H^(***q***_0_^R^) – *E*_T1_^H^(***q***_0_^H^)} being the geometrical effect and Δ*E*_S1–T1_^D^ = α_R(S1)_ – α_R(T1)_ being the zeroth-order differential effect of the substituent.

Hence, in this study, we will evaluate and rationalize the geometrical and differential effects of different chemical substituents on the modulation of the S_1_–T_1_ energy difference.

From a computational point of view, the geometrical effect is calculated using the following procedure: (i) optimization of the T_1_ energy minimum of the 33 derivatives under study (Chr-R structure in Figure 1c); (ii) replacement of the substituent, −NH_2_, −NO_2_, or −CH_3_, by an H atom, followed by T_1_ geometry optimization keeping frozen all the chromophore’s atoms (Chr-H structure in Figure 1b); (iii) computation of the S_1_ energy for both structures calculated at steps (i) and (ii) through a single-point calculation; (iv) calculation of the geometrical effect, Δ*E*_S1–T1_^G^. Using eq 11, Δ*E*_S1–T1_^D^ can be easily calculated as the difference between ΔΔ*E*_S1–T1_^R^ and Δ*E*_S1–T1_^G^.

A similar procedure based on the same principles can be followed to investigate the substituent effect on the modification of the emission color, that is, the change of the energy gap between the lowest excited and ground singlet states, ΔΔ*E*_S1–S0_^R^, this time considering the S_1_-optimized geometry as the starting structure. The detailed theoretical development is given in Section 2 of the Supporting Information (eqs S1–S9). Hence, similarly to eq 11, the modulation of the emission color induced by the introduction of a certain substituent can be divided into its geometrical and differential effects:
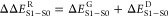
12

## 3 Results and Discussion

In this section, we will rationalize the obtained results in terms of the substituent effect to enhance the rISC efficiency and to possibly modulate the emission color of the PTZ-DBTO2 compound. For this aim, the section will be divided into five parts: (i) analysis of the properties of the reference system, i.e., unsubstituted PTZ-DBTO2 (see Scheme 1c); (ii) selection of substituents and of their positions to generate PTZ-DBTO2 derivatives; (iii) rationalization of the substituent effect concerning the S_1_–T_1_ energy difference, affecting directly the rISC efficiency; (iv) concerning the S_1_–S_0_ energy difference, responsible of the emission color modulation; (v) evaluation of the combined effect, i.e., both S_1_–T_1_ and S_1_–S_0_ energy differences, depending on the substitution pattern.

### 3.1 Reference System

**Figure 2:**
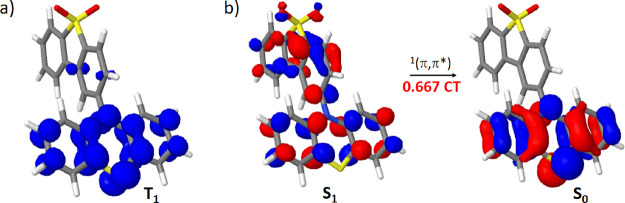
(a) T_1_ spin density and (b) natural transition orbitals involved in the S_1_ → S_0_ emissive transition, all computed for the T_1_ minimum geometry.

To understand the substituent effect, we first have to fully characterize and study the reference system, the unsubstituted PTZ-DBTO2. We start analyzing the properties of this system in the T_1_ minimum, from where the rISC takes place. For PTZ-DBTO2 and similar derivatives, it is known that two conformations are possible, the equatorial and axial conformations (Figure S1 of the Supporting Information).^14^ Hence, we have optimized both conformations in T_1_, finding that the equatorial one is slightly more stable (ca. 0.1 eV) than the axial conformation. For this reason, we have selected the equatorial conformation to further analyze its properties in T_1_. As aforementioned, one of the factors governing the rISC efficiency is the S_1_–T_1_ energy difference at the T_1_ minimum (Scheme 1b). In the case of the reference system, we have defined such energy difference as Δ*E*_S1–T1_^H^(***q***_0_^H^) and its computed value is 5.07 kcal/mol (0.22 eV). This value is certainly far from energy degeneration, corresponding to the optimal condition, and it is also larger than the 4.6 kcal/mol (0.20 eV) limit established in previous works for ensuring efficient rISC in this type of systems.^14,21,28−31^ Hence, there is certainly room for improvement through substituted derivatives. As previous studies related Δ*E*_S1–T1_ with the electronic nature of these two states, we have characterized them for this reference system at the T_1_ minimum geometry. In particular, the analysis of the T_1_ spin density let us conclude that T_1_ is an LE state, as the spin density is mainly located in the donor moiety: the spin density index of the donor moiety (SD_donor_) is 0.90 (Figure 2a). In contrast, by analyzing the natural transition orbitals describing the S_1_ state, at the same T_1_ minimum geometry, we can conclude that S_1_ is a CT state, as compared to S_0_ (Figure 2b). In fact, we have performed a quantification of this CT character, finding that the electronic density is transferred between the donor and the acceptor moieties (see Scheme 1c for their definition) with a CT index of 0.667 (1.0 would represent a total CT of one electron between the donor and acceptor moieties). The concluded electronic nature of the T_1_ and S_1_ states is in agreement with previous computational studies of this system.^14,23,31,74^ In the energetic point of view, we found in literature that usually Δ*E*_S1–T1_ is considered at the S_1_ minimum, corresponding in our case to 0.7 kcal/mol (0.03 eV), in good agreement with experimental (0.02 eV^52^ and 0.05 eV^41^) and computational (0.02 eV)^31^ published results.

The second aim of this work is to modulate the color emission, that is, Δ*E*_S1–S0_, through a substituent effect. For this reason, we have optimized the equatorial conformation in S_1_: at the S_1_ minimum, *E*_S1–S0_ = 60.9 kcal/mol (2.64 eV, 469 nm), in agreement with the already reported values computed at the same level of theory.^14^ It should be noted that the electronic nature of S_1_ at its minimum is kept as a CT state but its CT character is increased, corresponding to the almost quantitative exchange of one electron between donor and acceptor moieties (CT index of 0.943, Figure S2 of the Supporting Information).

### 3.2 Substituted Derivatives

**Figure 3:**
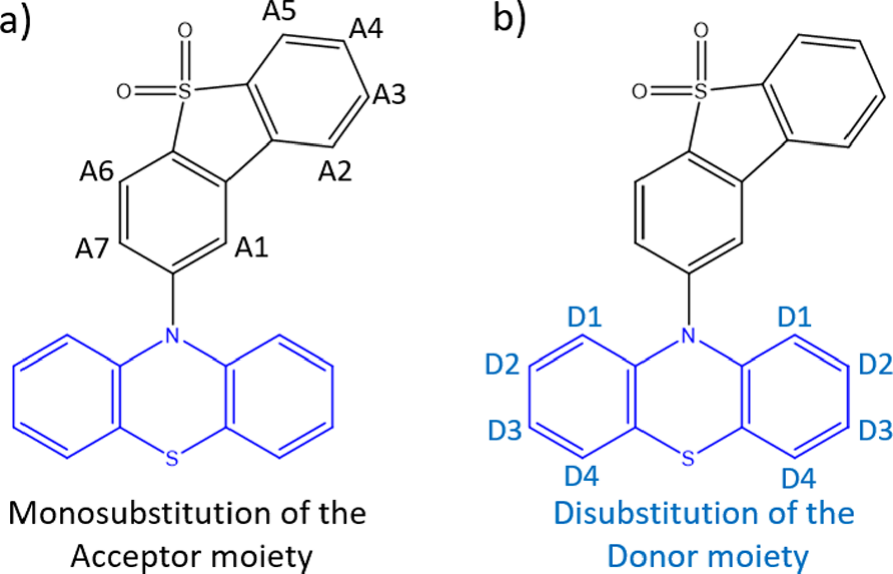
(a) Positions in the acceptor moiety (depicted as A1–A7) leading to monosubstituted derivatives and (b) positions in the donor moiety (depicted as D1–D4) leading to disubstituted derivatives.

With the aim of improving the rISC efficiency of the PTZ-DBTO2 reference system (reaching the condition Δ*E*_S1–T1_≈ 0 eV) and shifting its emission color (decreasing or increasing Δ*E*_S1–S0_), we have proposed specific substitution patterns, concerning both the substitution position and the electronic nature of the substituent. Regarding the substitution positions, we can discern two groups of derivatives. The first group includes monosubstitutions of the acceptor moiety, i.e., dibenzothiophene-*S*,*S*-dioxide (Figure 3a, positions A1–A7), while the second group includes disubstitutions in the donor moiety, i.e., phenothiazine (Figure 3b, positions D1–D4). Indeed, as it can be seen in Figure 2, the acceptor moiety is perpendicular to the donor one; hence, the D1–D4 positions are geometrically equivalent with respect to the acceptor. To achieve a more pronounced substituent effect, we have therefore decided to disubstitute the donor.

Concerning the electronic nature of the substituent, we have selected a strong electron-donating group (amine group, −NH_2_), a strong electron-withdrawing group (nitro group, −NO_2_), and a group with no significant electronic effect (methyl group, −CH_3_). Each group has been placed in the substitution positions depicted in Figure 3, leading to a total of 33 PTZ-DBTO2 derivatives (see each chemical structure in Figure S3 of the Supporting Information). For clarity, each derivative will be denoted considering the substituent and its position, e.g., a derivative with one methyl group in position A2 will be denoted as CH_3_-A2. Thanks to the wide variety of substituted derivatives under study, we can therefore rationalize both the electronic nature and the position of the substituent effect on the rISC efficiency and color emission.

### 3.3 Modulation of the rISC Efficiency by Substitution

As a reminder, the increase of the rISC efficiency in TADF processes is a key aspect to develop better OLEDs, as the more efficient the rISC is, the higher is the population of S_1_, thus allowing fluorescence. The rISC efficiency increases as long as the energy difference between the two states (Δ*E*_S1–T1_) decreases. In this section, we are going to rationalize how Δ*E*_S1–T1_ varies depending on the substitution pattern, paying special attention to the cases in which the S_1_–T_1_ energy difference is between 0 and 4.6 kcal/mol (0.20 eV).^14^ For this aim, and as described in Section 2, we apply a theoretical model to get insight into the substituent effect by analyzing separately two factors: (i) the geometrical effect, intended as the geometrical modification of the chromophore coordinates provoked by substitution, compared to the unsubstituted system, and (ii) the differential effect, which includes the electronic contribution of the specific substituent taken into account and its interaction with the chromophore.

#### 3.3.1 The Role of the Geometrical Effect on rISC Efficiency

**Figure 4:**
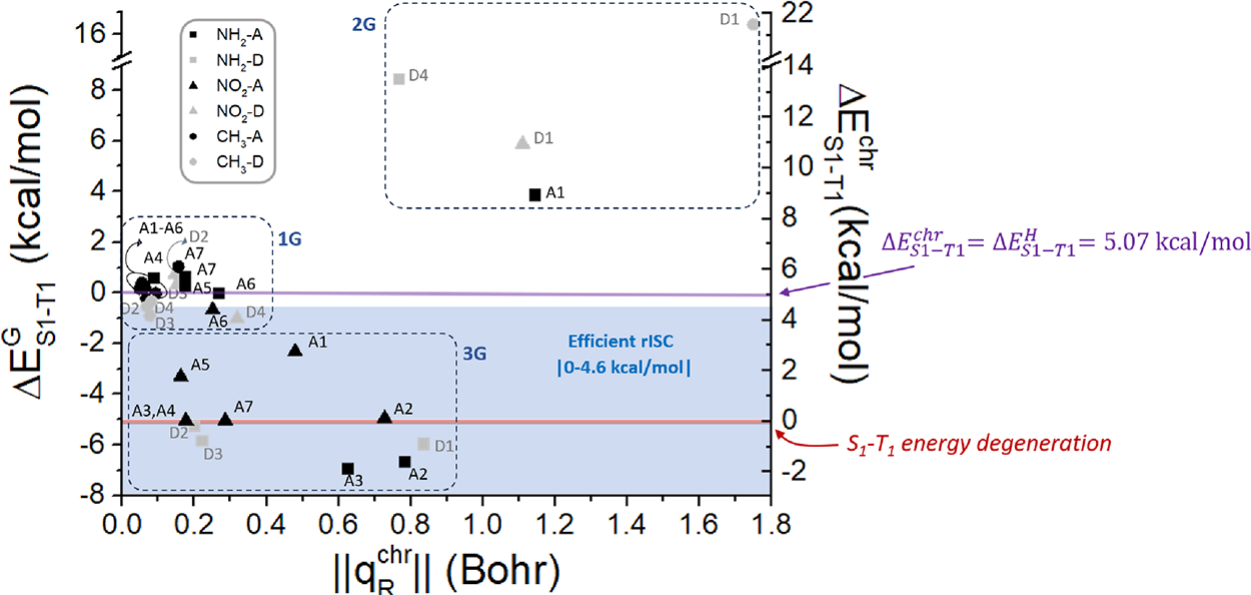
Representation of the geometrical effect on the S_1_–T_1_ energy difference (Δ*E*_S1–T1_^G^, left *y*-axis) and the S_1_–T_1_ energy gap for the H-substituted form of the derivative (Δ*E*_S1–T1_^chr^, right *y*-axis) computed at the T_1_ minimum, against the module of the displacement of the chromophore’s coordinates due to substitution (∥***q***_R_^chr^∥ ≡ ∥***q***_0_^R^*–**q***_0_^H^∥). The three groups of derivatives 1G, 2G, and 3G are shown, based on the range of Δ*E*_S1–T1_^G^.

In more detail, concerning the geometrical effect, we have calculated the modulation of Δ*E*_S1–T1_ due to the modification of the chromophore’s coordinates after substitution (Δ*E*_S1–T1_^G^), following the procedure described in Section 2. It should be recalled that this geometrical effect neglects the effect of substitution on the electron density, considering only the purely geometrical distortion of the chromophore. The results are presented in Figure 4, based on both the energy gap variation (Δ*E*_S1–T1_^G^, left *y-*axis) and the energy gap value considering only the chromophore’s distortion (*E*_S1–T1_^H^, right *y-*axis), which are linearly related by eqs 8 and 9: Δ*E*_S1–T1_^G^ = Δ*E*_S1–T1_^H^(***q***_0_^R^) – Δ*E*_S1–T1_^H^(***q***_0_^H^), where Δ*E*_S1–T1_^H^(***q***_0_^H^) = 5.07 kcal/mol for PTZ-DBTO2. Such energy gaps are represented as functions of the structural differences between the geometry of the reference molecule (PTZ-DBTO2) and the geometry of the H-substituted (−NH_2_, −NO_2_, or −CH_3_ replaced by −H) compounds obtained for the 33 derivatives. Each geometrical difference is defined as a displacement (∥***q***_R_^chr^∥ ≡ ∥***q***_0_^R^*– **q***_0_^H^∥, *x*-axis in Figure 4). At a first sight, we can just observe a large variability, not easily finding any trend regarding either the substituent nature or the substituent position. Nevertheless, by analyzing more carefully the data of Figure 4, we can sort all derivatives into three groups: (i) group 1G, including the derivatives keeping almost invariant the S_1_–T_1_ energy gap, i.e., ca. 5.07 kcal/mol in terms of *E*_S1–T1_^chr^, corresponding to an almost null geometrical effect (Δ*E*_S1–T1_^G^ between −1 and 1 kcal/mol); (ii) group 2G, including the derivatives characterized by a consistently positive geometrical effect (Δ*E*_S1–T1_^G^≥1 kcal/mol), thus increasing *E*_S1–T1_^chr^ and hence worsening the rISC efficiency; (iii) group 3G, including derivatives with a consistently negative geometrical effect Δ*E*_S1–T1_^G^≤1 kcal/mol, thus decreasing Δ*E*_S1–T1_^chr^ and keeping it within the |0–4.6 kcal/mol| range considered for efficient rISC, in some cases reaching the desired S_1_–T_1_ energy degeneracy (Δ*E*_S1–T1_^G^ ≈ −5.07 kcal/mol, Δ*E*_S1–T1_^chr^≈ 0 kcal/mol) required for optimal rISC.

If we pay attention to the derivatives included in each group (1G, 2G, and 3G), the following general trends can be found: regarding group 1G, it is formed by NH_2_–(A4–A7), NO_2_–(A6, D2–D4), and all methylated derivatives (with the exception of CH_3_–D1 that will be later discussed). This group is characterized by small modules of the displacement vector, that is, the chromophore geometry is only slightly modified due to substitution (<0.4 Bohr). Group 2G, decreasing rISC efficiency, includes only four derivatives, three of them corresponding to substitution placed between the acceptor and donor moieties (D1 and A1 positions), possibly leading to steric hindrance. In fact, the module of the displacement vector for these four derivatives is quite large (0.8 < ∥***q***_R_^chr^∥< 1.8 Bohr), compared to the other compounds under study, pointing to a large modification of the chromophore structure due to substitution. Finally, group 3G improves rISC efficiency, including almost all NO_2_–A derivatives (except NO_2_–A6), together with NH_2_–(A2,A3,D1–D3), for a total of 11 derivatives, i.e., one-third of the studied derivatives.

**Figure 5:**
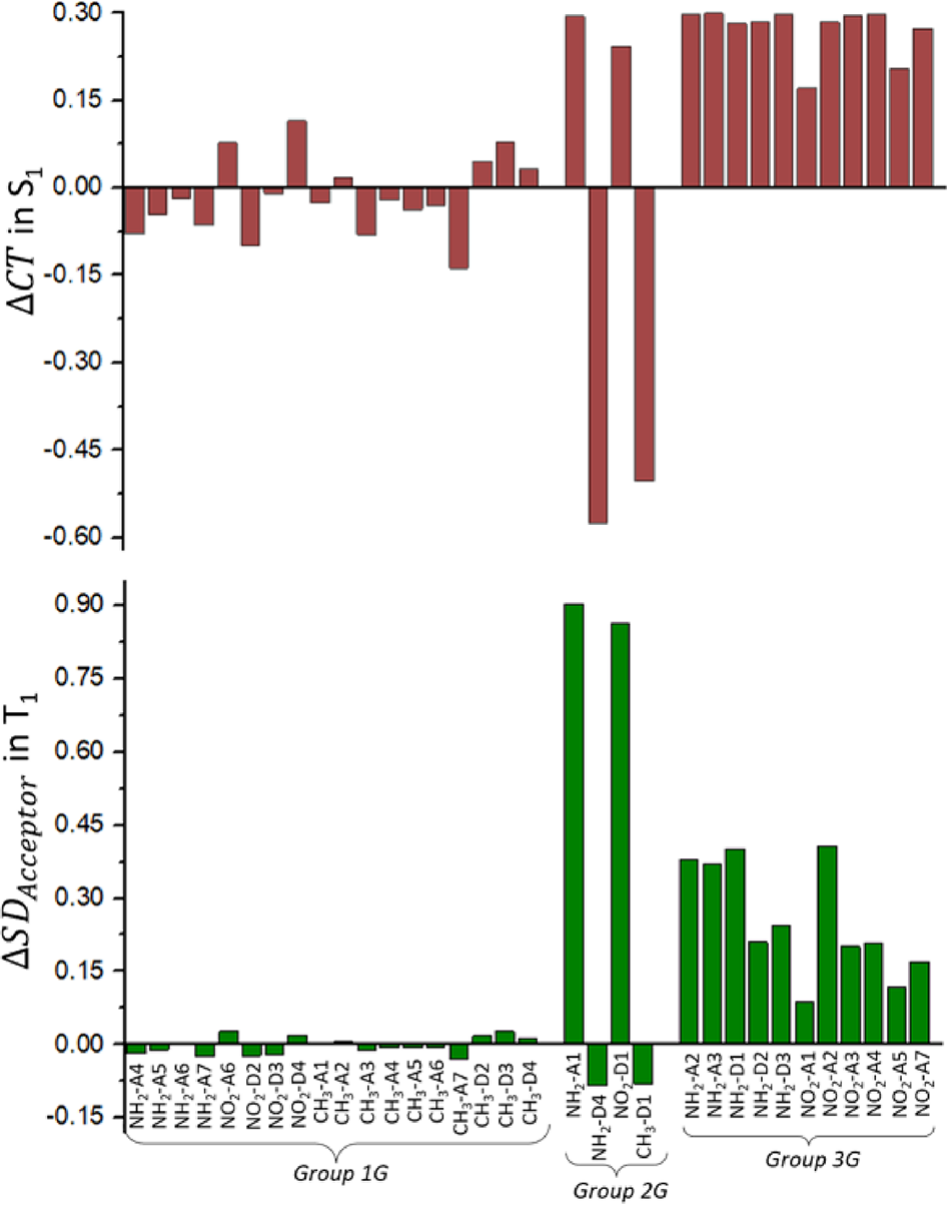
Variation of the S_1_ CT and of the T_1_ SD_acceptor_ indexes for the derivatives under study, evaluated in the T_1_ energy minimum, with respect to the reference molecule, affecting the geometrical effect.

In any case, reasoning on the position or electronic nature of the substituent, it is still difficult to assign a given derivative to one of these three groups. We have therefore analyzed different properties of all derivatives. First, we have plotted Δ*E*_S1–T1_^G^ against the variation of the S_1_ CT index (Figure S4): an almost linear dependence, showing a Δ*E*_S1–T1_^G^ increase as long as the CT character of S_1_ decreases, was found for all derivatives but NO_2_–D1 and NH_2_–A1, pointing to additional properties we are not considering. Then, we have analyzed simultaneously the variation of the CT and SD_Acceptor_ indexes, looking for the variation of the T_1_ electronic nature, rarely taken into account to describe the TADF process in OLEDs.^39^ Our reference PTZ-DBTO2 is characterized by an S_1_ state of CT character (%CT = 0.667) and a T_1_ state of LE character, with the spin density localized in the donor moiety (SD_donor_ = 0.90, Figure 2). To check for the geometrical modification provoked by the substituent, the CT index of S_1_ and the SD_Acceptor_ index of T_1_ have been computed for the H-substituted T_1_ minima of the derivatives, i.e., the relaxed structures with the substituent replaced by a hydrogen atom (see Section 2). The results are shown in Figure 5: positive (negative) values indicate an increase (decrease) of the CT or SD_acceptor_ indexes. In group 1G, the geometrical modification of the chromophore due to substitution does not have a relevant impact on the CT character of S_1_ (ΔCT < 0.14) neither on the SD_acceptor_ of T_1_ (Δ*S*D_acceptor_ < 0.03). In this case, Δ*E*_S1–T1_^G^ ≈ 0 and the S_1_–T_1_ energy gap remain close to the one of the reference.

Regarding group 2G, we observe two different behaviors. On the one hand, NH_2_–A1 and NO_2_–D1 (the two aforementioned outliers in the linear trend between Δ*E*_S1–T1_^G^ and ΔCT) result in an increase of the S_1_ CT character by ca. 0.3, reaching almost quantitative CT from the donor to the acceptor moieties (CT index = 0.667 + ∼0.3 ≈ 1). Moreover, the geometrical distortion of the chromophore for these two derivatives has a significant effect on the T_1_ electronic nature as for both, T_1_ is a LE state with the spin density localized in the acceptor moiety instead of the donor moiety, as it was in the case of the reference (SD_acceptor_ index = (1 – SD_Donor_) + ΔSD_acceptor_ = (1–0.9) + ∼0.9 ≈ 1), thus corroborating our hypothesis that, for these two derivatives, the T_1_ electronic nature is an additional property to be considered together with the S_1_ electronic nature. On the other hand, the geometrical effect of NH_2_–D4 and CH_3_–D1 has a clear impact only on the S_1_ electronic nature, resulting in both cases in an LE state (CT index = 0.667 to ∼0.6 ≈ 0) while keeping the electronic nature of T_1_ essentially unchanged. Hence, we can conclude that both the change of the T_1_ electronic nature to an LE localized in the acceptor moiety and the change of the S_1_ nature to an LE state would lead to an increase of the S_1_–T_1_ energy gap (more pronounced in the second case), hampering the rISC efficiency.

Finally, group 3G results in all cases in an enhanced S_1_ CT character (CT index ≥0.84). Moreover, the T_1_ spin density of the derivatives belonging to group 3G is not anymore located either in the donor moiety (as for the reference and group 1G) or in the acceptor moiety (as for group 2G) but is delocalized all along the molecule with a slight preference for the donor moiety (a value of SD_donor_ or SD_acceptor_ of 0.5 means an equal delocalization of the spin density in the acceptor and donor moieties).

Considering that the physical interest is focused on group 3G (including the derivatives allowing an increase in rISC efficiency), we have further analyzed it in terms of chromophore geometrical distortion: S_1_–T_1_ energy degeneracy can be obtained by small distortions, ∥***q***_0_^R^*–**q***_0_^H^∥ ≡ | | ***q***_R_^chr^ | |= 0.2 to 0.8 Bohr (Figure 4), corresponding to intermediate (0.16 to 0.25) and largest (>0.37) ΔSD_acceptor_ values (Figure 5).

Therefore, it can be concluded that delocalization of the T_1_ spin density among the acceptor and donor moieties due to the geometrical effect is the most relevant property leading to S_1_–T_1_ rISC efficiency increase. The optimal condition (S_1_–T_1_ energy degeneracy) can be accomplished, in terms of the geometrical effect, for compounds NO_2_–(A2–A4,A7) and NH_2_–D2. In some cases, the relative stability of S_1_ and T_1_ is reversed (NH_2_–A2,A3,D1,D3) while keeping an energy gap within the 4.6 kcal/mol limit considered for efficient rISC.^14^

#### 3.3.2 The Role of the Differential Effect on rISC Efficiency

**Figure 6:**
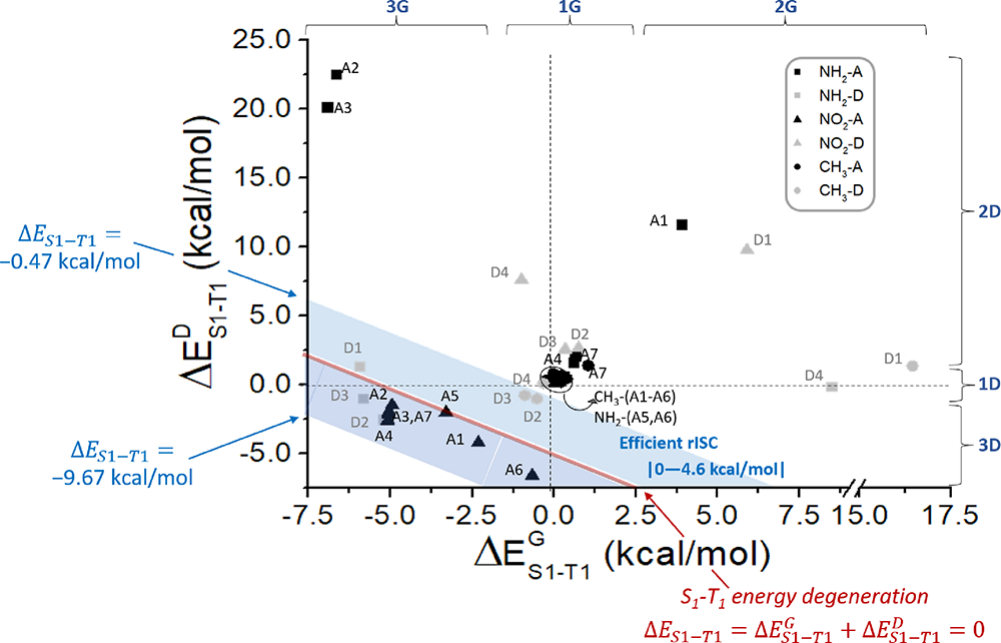
Representation of the differential effect vs the geometrical effect on Δ*E*_S1–T1_ for the derivatives under study, computed at the T_1_ minimum. The axes passing through their origin are depicted by dotted lines. The three groups of derivatives 1D/2D/3D and 1G/2G/3G are shown, based on the respective energy ranges, as well as the linear equations defining S_1_–T_1_ energy degeneration (red line), upper and lower boundaries of the efficient rISC zone (light blue area).

The differential effect Δ*E*_S1–T1_^D^, which includes the electronic effect, is shown vs the geometrical effect Δ*E*_S1–T1_^G^ in Figure 6. Depending on the modulation, we can classify the compounds into three groups: (i) group 1D corresponding to the derivatives for which the differential effect is almost negligible (≤±1 kcal/mol), including NH_2_–(A5,A6,D1,D3,D4), CH_3_–(A1–A6,D1–D4), and NO_2_–A2; (ii) group 2D, characterized by a consistently positive differential effect, increasing Δ*E*_S1–T1_ and including NH_2_–(A1–A4,A7), NO_2_–(D1–D4), and CH_3_–A7; (iii) group 3D, including compounds showing a negative differential effect, decreasing Δ*E*_S1–T1_ and formed by NH_2_–D2 and NO_2_–(A1,A3–A7).

**Figure 7:**
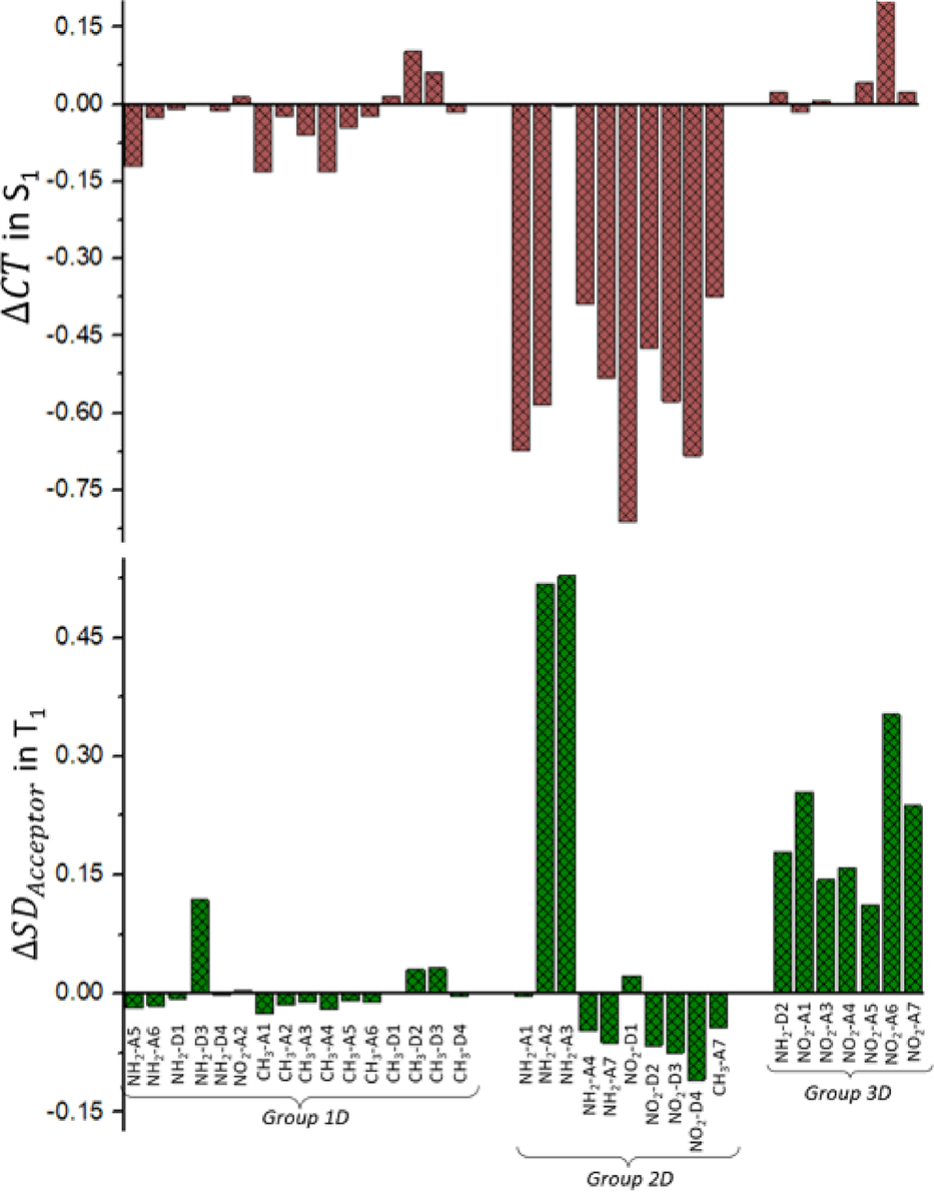
Variation of the S_1_ CT and of the T_1_ SD_acceptor_ indexes for the derivatives under study, evaluated in the T_1_ energy minimum, with respect to the geometrical effect, affecting the differential effect.

In this case as well, we have analyzed different properties with the goal of understanding these three different behaviors. First, we have plotted Δ*E*_S1–T1_^D^ as a function of the variation of the S_1_ CT index (Figure S5). Similarly to the geometrical effect, a linear trend, characterized by Δ*E*_S1–T1_^D^ increasing as long as the S_1_ CT character decreases, is found with the exception of some outliers. Indeed, also for the differential effect, changes in the S_1_ CT character and in the T_1_ SD localization are the key properties explaining the different Δ*E*_S1–T1_^D^ values observed. In Figure 7, we depict the variations of the CT and SD indexes, although this time with respect to the geometrical effect.

The results obtained can be rationalized as follows: for group 1D, we observe a limited modulation of both S_1_ CT and T_1_ SD_acceptor_ indexes (<±0.15), therefore resulting in an almost insignificant differential effect on Δ*E*_S1–T1_.

Regarding group 2D, we observe a general significant decrease of the S_1_ CT character (except for NH_2_–A3), changing the electronic nature of this state to an LE one. However, two patterns are observed for the modulation of the T_1_ SD_acceptor_ index within this group: a limited modulation is observed (similarly to group 1D, <±0.15) for NH_2_–(A1,A4,A7), NO_2_–(D1–D4), and CH_3_–A7, while a significant increase (ca. 0.5) is observed for NH_2_–(A2,A3). If we analyze the extent of the differential effect for the latter two compounds in Figure 6, we note that it is much more pronounced (>20 kcal/mol, >0.87 eV) than the rest of compounds of this group, indeed corresponding to the outliers found in the linear trend of Figure S5. This shows once more the crucial role of the T_1_ electronic nature in modulating the rISC efficiency.

Finally, group 3D is characterized by a limited change of the S_1_ CT character, similarly to group 1D, keeping the electronic nature imposed by the geometrical effect, except for NO_2_–A6, characterized by a larger differential effect (see Figure 6). On the contrary, a relevant increase of the SD_acceptor_ index is observed.

All in all, we have been able to rationalize the characteristic properties of 1D, 2D, and 3D groups leading to diverse differential effects: group 1D is almost not affected, while a positive effect is in general due to a decrease of the S_1_ CT character (group 2D) and a negative effect is produced by an increase of the spin density localized in the acceptor part (group 3D).

#### 3.3.3 Combining Geometrical and Differential Effects on rISC Efficiency

To discern if a particular substitution pattern can reach the aim of increasing the rISC efficiency by decreasing Δ*E*_S1–T1_, both geometrical and differential effects need to be considered. For this purpose, Figure 6 can be again very helpful, since it plots Δ*E*_S1–T1_^D^ as a function of Δ*E*_S1–T1_^G^ for the 33 compounds under study, hence offering the opportunity to show the combined effect. Indeed, analogously to Figure 4, also in Figure 6, a light-blue area is depicted defining the efficient rISC zone, containing a red line for which the S_1_–T_1_ energy degeneration condition is fulfilled, by applying eq 11: Δ*E*_S1–T1_ = Δ*E*_S1–T1_^G^ + Δ*E*_S1–T1_^D^ = 0. To show its validity, we can easily find in Figure 6 the value of Δ*E*_S1–T1_^G^ when Δ*E*_S1–T1_^D^ = 0, corresponding to −5.07 kcal/mol to fulfill S_1_–T_1_ energy degeneration. This value is the same that we can observe on the *y*-axis of Figure 4, i.e., when only the geometrical effect (and hence no differential effect) was considered. As previously defined, the efficient rISC zone depicted in Figure 6 covers, as in Figure 4, an area corresponding to ±4.6 kcal/mol starting from the S_1_–T_1_ energy degeneration condition. In Figure 6, all possible combinations of Δ*E*_S1–T1_^G^ and Δ*E*_S1–T1_^D^ are taken into account, and by simple math, we can calculate the equations defining upper and lower boundaries of such efficient rISC zone: Δ*E*_S1–T1_^G^ + Δ*E*_S1–T1_^D^= −0.47 kcal/mol and Δ*E*_S1–T1_^G^ + Δ*E*_S1–T1_^D^= −9.67 kcal/mol, respectively.

**Figure 8:**
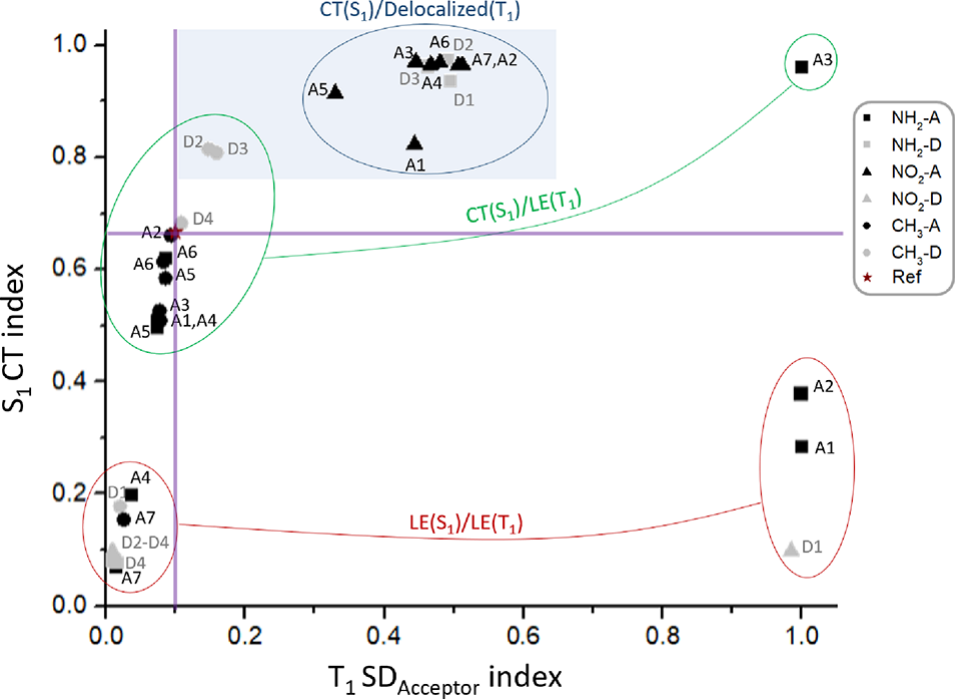
Representation of the S_1_ CT index as a function of the T_1_ SD_acceptor_ index, calculated at the T_1_ minimum for each compound under study. The purple lines indicate the index values of the PTZ-DBTO2 reference system. The derivatives are grouped into three classes, depending on the electronic nature of their S_1_ and T_1_ states. The compounds belonging to the efficient rISC zone are highlighted by a light-blue area.

In Figure 8, we show the S_1_ CT index as a function of the T_1_ SD_acceptor_ index. Within the efficient rISC zone, except for CH_3_–D2,D3, S_1_ is a CT state, with an increased CT character compared to the reference compound, and T_1_ is characterized by a delocalization of its spin density (blue circle in Figure 8). Instead, for CH_3_–(D2,D3), S_1_ is again a CT state, but in T_1_, the spin density is mainly localized on the donor moiety (SD_acceptor_ ≅ 0.15; SD_donor_ ≅ 1 – SD_acceptor_ = 0.85). The rest of the compounds, falling out of the efficient rISC zone, can follow two distinctive scenarios: on the one hand, as well as CH_3_–(D2,D3), a group of derivatives keeps a mainly S_1_ CT character, especially for NH_2_–A3, while an LE state is found for *T*_1_, localized in the donor moiety for all compounds but NH_2_-A3 (green circles in Figure 8). On the other hand, another group of derivatives is characterized by S_1_ and T_1_ LE states (red circles in Figure 8).

Hence, we can conclude that, in order to enhance the rISC efficiency of the PTZ-DBTO2 system, the substitution pattern should be able to increase the S_1_ CT character of the reference compound and, at the same time, the T_1_ spin density should be delocalized along the molecule. Compounds for which S_1_ is an LE state or in T_1_ the spin density is localized in one part of the molecule are expected to lower their rISC efficiency. This interpretation is in agreement with the already published four-state model^39^ (Figure S6).

With all the collected information and the elaborated results, we can finally discuss about the substitution patterns expected to improve the rISC efficiency and, therefore, the TADF process: placing the −NH_2_ group in any of the acceptor moiety positions or the −NO_2_ group in the D1–D3 donor moiety positions should result in an efficient rISC. Hence, introducing an electron-donating group in the acceptor moiety or an electron-withdrawing group in the donor moiety should decrease Δ*E*_S1–T1_. This conclusion can be counterintuitive, since chemical intuition should lead to the reasoning that, to increase the S_1_ CT character, electron-donating groups should be substituents of the donor moiety and electron-withdrawing groups should be substituents of the acceptor moiety while the opposite is interestingly found by applying our methodology.

In addition, we have computed the SOC values for the reference molecule and the 33 derivatives under study, as SOC is also related to the rISC efficiency. For the reference system, a small SOC value of 0.40 cm^–1^ has been computed, as expected for this type of organic compounds.^17,43^ Substitution has not a large impact on the SOC values (Table S4), keeping the same order of magnitude, with the exception of NH_2_–D4 and CH_3_–D1 for which it is one order of magnitude larger. This means that, within the limits of our level of theory, S_1_ and T_1_ states are not characterized by a remarkable change in the nature of the electronic configuration, even when PTZ-DBTO2 is substituted with NO_2_, although in other chromophores NO_2_ increases considerably the SOC.^75^

### 3.4 Modulation of the Emission Energy by Substitution

**Figure 9:**
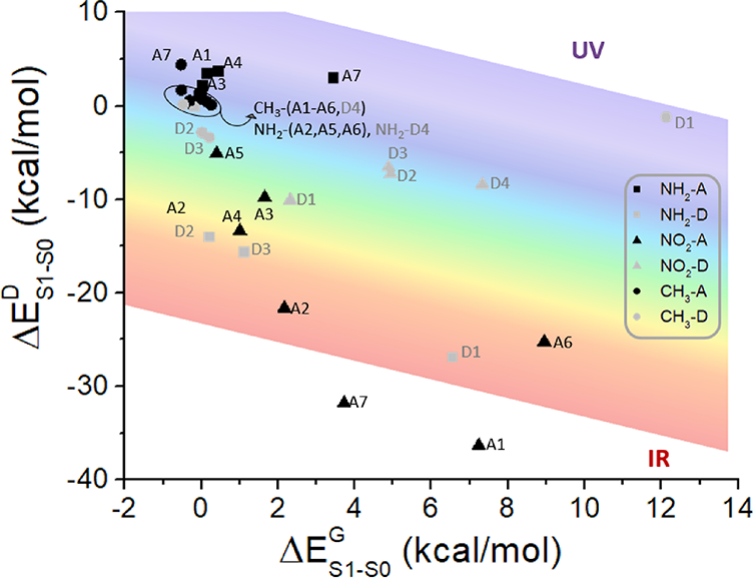
Representation of the differential effect vs the geometrical effect on the emission color modulation (Δ*E*_S1–S0_), for each of the proposed substituents of PTZ-DBTO2. UV, visible, and IR spectral ranges are depicted.

As shown in Scheme 1b, once S_1_ is populated, fluorescence can follow. If fluorescence is within the visible spectrum range, such photon emission corresponds to a color, depending on the S_1_–S_0_ energy difference. Evidently, the emission color is a key property for OLED applications, since there is no technological interest in increasing the rISC efficiency for a compound that lately emits in an unwashed color range or, even worse, out of the visible-spectrum range (UV or infrared windows). For this reason, it is mandatory to explore the substituent effect also on the emission color modulation, Δ*E*_S1–S0_. The results, analogously to Figure 6 for Δ*E*_S1–T1_, are presented in Figure 9 by a graph showing Δ*E*_S1–S0_^D^ vs Δ*E*_S1–S0_^G^. In this context, the reference value is *E*_*S*1–*S*0_ calculated at the S_1_ minimum geometry for PTZ-DBTO2:60.9 kcal/mol (469 nm, 2.64 eV), corresponding to cyan.

At a first glance, Figure 9 suggests that Δ*E*_S1–S0_ can be easily modulated by substitution or blue-shifting or red-shifting the emission color.

Concerning the geometrical effect, it is negligible (≤±1 kcal/mol) for 20 of the 33 derivatives under study, with the largest positive effects mainly found for the −NO_2_ derivatives. Similarly to the analysis of the rISC efficiency (Figure 4), we have first examined the dependence of Δ*E*_S1–S0_^G^ from the modulus of the chromophore’s structure displacement (Figure S7), also in this case not identifying a clear trend. With the aim of understanding the observed largest geometrical effects—including the 11 derivatives for which Δ*E*_S1–S0_^G^ is larger than 2 kcal/mol (0.09 eV)—we have first evaluated the variation of the S_1_ CT index (Figure S8): 8 of these 11 derivatives are characterized by a relevant decrease of the S_1_ CT character, leaving out NH_2_–D1 and NO_2_–(A2,A7). Nevertheless, a full explanation can be found by computing the S_1_ and S_0_ energy variation due to the geometrical effect (Figure S9). Note that while the S_0_ energy could be in principle stabilized or destabilized by substitution, the S_1_ energy can be only destabilized, since our reference is placed at its S_1_ minimum and, therefore, any displacement results in an increase of the S_1_ energy, since we are approximating our PESs by a quadratic expansion centered at the energy minimum structure. Looking at Figure S9, we observe that while S_0_ is significantly destabilized (Δ*E*_S0_ > 2 kcal/mol) only for compounds (NH_2_,NO_2_,CH_3_)–D1, S_1_ is significantly destabilized (Δ*E*_S1_ > 2 kcal/mol) for all the 11 derivatives showing a pronounced geometrical effect. In any case, the differential effect has a larger impact on the emission color modulation (Figure 9). Indeed, while Δ*E*_S1–S0_^G^ spans a range between −1 and +12.5 kcal/mol, Δ*E*_S1–S0_^D^ covers a range from −36 to +7 kcal/mol. Hence, the geometrical effect mainly accounts for a limited blue shift, while the differential effect can explain a consistent red shift, reaching the infrared (IR) spectral region, with 13 out of the 33 derivatives decreasing Δ*E*_S1–S0_ more than 6.5 kcal/mol. It should be remarked that these 13 derivatives include almost all the −NO_2_ derivatives and three −NH_2_ derivatives. We have further analyzed their natural transition orbitals involved in the S_1_ → S_0_ emission process, finding a common characteristic: the substituent participates in the electronic transition, being located part of the electron density in the corresponding substituent (Figure S10).

To discern among the different derivatives, we can explain separately the results obtained by introducing the electron-withdrawing −NO_2_ group or the electron donor −NH_2_ group: compounds NO_2_–(A1–A7), corresponding to substitution of all possible positions in the acceptor moiety, show a wide range of differential effects, all negative Δ*E*_S1–S0_^D^ values. As it can be seen in Figure 9, the closer to the donor moiety the position occupied by the −NO_2_ group, the more negative the Δ*E*_S1–S0_^D^ value, reaching −36 kcal/mol (−1.56 eV) for NO_2_–A1. On the other hand, when occupying the furthest position from the donor moiety, the −NO_2_ group induces the smallest differential effect: −6 kcal/mol (0.26 eV) for NO_2_-A5. In general, we can state that the inclusion of the electron-withdrawing −NO_2_ substituent in the dibenzothiophene-*S*,*S*-dioxide moiety, increases the acceptor character of this part of the molecule. On the contrary, when including the −NO_2_ substituent in the donor moiety, the differential effect is much narrower, although again negative (between −7 and −10 kcal/mol for NO_2_–(D1–D4)). In these cases, the substituent is involved in the S_1_ → S_0_ emissive transition but the electronic nature of S_1_ changes, leading to an LE state, since the −NO_2_ group competes as acceptor with the dibenzothiophene-*S*,*S*-dioxide acceptor moiety (Figure S10).

For the electron donor −NH_2_ substituent, the same reasoning followed for the −NO_2_ substituent can be applied: the most consistent differential effect can be observed when −NH_2_ is a substituent of the phenothiazine donor moiety, strengthening the CT character of the S_1_ → S_0_ transition. Indeed, the NH_2_–(D1–D4) compounds allow to span a Δ*E*_S1–S0_^*D*^ range between 0 and −26 kcal/mol, with the highest effect observed when substituting the nearest position to the acceptor moiety (D1) and the lowest effect obtained when substituting the furthest position from the acceptor moiety (D4), which indeed is not involved in the emission transition (Figure S10). On the contrary, when placing the −NH_2_ substituent in the acceptor moiety, resulting in NH_2_–(A1–A7) compounds, the Δ*E*_S1–S0_^D^ values become positive and lie in a narrow range (0–3 kcal/mol).

Bearing in mind the most consistent Δ*E*_S1–S0_^D^ effect compared to Δ*E*_S1–S0_^G^, in terms of energy ranges, we could conclude that differential (electronic) effects are predominant over the geometric effects.

Considering both geometrical and differential effects altogether, if we are interested in a fluorescence red-shift, it can be achieved by either (i) introducing the −NO_2_ substituent in the acceptor moiety (black triangles in Figure 9) or (ii) introducing the −NH_2_ substituent in the donor moiety (gray squares, except NH_2_–D4), whereas, if we are interested in a (slight) fluorescence blue shift, it can be achieved (i) by introducing the −NH_2_ substituent in the acceptor positions A1, A3, A4, and A7 or (ii) for CH_3_–D1 due to the large steric hindrance caused by the methyl group.

Apart from the shift of the emission color, there is another property involved in the emission process that is regarded as key for OLED applications: the emission intensity. For this reason, we have computed the oscillator strengths of the S_1_ → S_0_ transition for all studied compounds. As a reminder, the oscillator strength is mainly due to the overlap between the orbitals involved in the electronic transition. Therefore, when CT states are involved, such overlap is small, thus leading to low oscillator strength values. However, we have already concluded that for this system, the rISC efficiency is enhanced (in some cases reaching the optimal degeneration of S_1_ and T_1_ energies) when S_1_ is a CT state and T_1_ has its spin density delocalized. This means that an enhancement of the rISC efficiency leads inevitably to a low value of the S_1_ → S_0_ oscillator strength. This should be reminded as an inherent limitation of these compounds and, more in general, of TADF emitters.^76^ Regarding the computed values, the oscillator strength of the reference system is quite low, 5 × 10^–4^. Compared to it, the oscillator strength is enhanced only for the NO_2_–(D_1_–D_4_) compounds (Figure S11), since they are characterized by the most pronounced S_1_ LE state (Figure 8). However, these compounds present large S_1_–T_1_ energy gaps (Figure 6), resulting in an inefficient rISC process.

### 3.5 Combined Modulation of the rISC Efficiency and Emission Energy by Substitution

**Figure 10:**
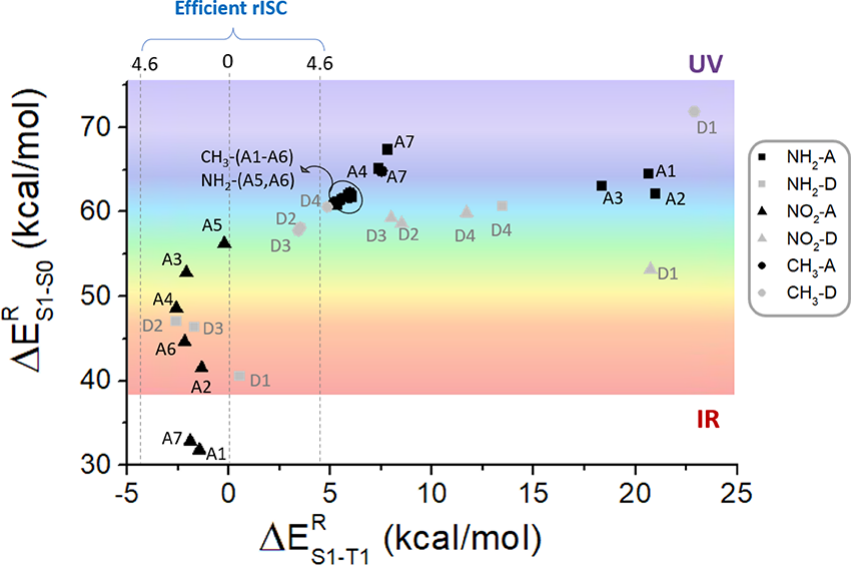
Representation of the S_1_–S_0_ emission energy gap vs the corresponding S_1_–T_1_ energy gap, computed for the 33 derivatives under study. UV, visible, and IR spectral ranges are depicted.

The best scenario for an efficient TADF emitter would be the one promoting an energy degeneracy of the S_1_–T_1_ energy gap, maximizing the rISC efficiency, while shifting the S_1_–S_0_ energy gap toward the desired color emission (see Figure S12 for details about rISC efficiency within the Marcus theory). In Figure 10, for each derivative, the S_1_–S_0_ energy gap is shown vs the corresponding S_1_–T_1_ energy gap: we can first conclude that, on the one hand, the derivatives offering an optimal rISC energy range (*E*_S1–T1_ in the range between −4.6 and +4.6 kcal/mol) lead in all cases to a red shift of the emission color. On the other hand, a blue shift involves a decrease of the rISC efficiency. This finding is a clear limitation of the class of compounds under study, in the case that blue emission is desired. If a red shift of the emission color is preferred, then specific substitution patterns of PTZ-DBTO2 can be suggested depending on the specific target color, moreover ensuring a suitable increase of the overall TADF process. Also, it should be noted that the inversion of S_1_ and T_1_ shown by several compounds (corresponding to negative Δ*E*_S1–T1_^R^ values in Figure 10)^77^ is highly sensitive to the calculation method used to obtain T_1_ energies: when using TD-DFT also for T_1_, the same trend is observed among the derivatives, but shifting the S_1_–T_1_ energy difference toward slightly more positive values, confirming the possibility to reach energy degeneracy, but not inversion (see Figure S13).

In particular, the case of NH_2_–D1 and NO_2_–(A1,A2) for which the rISC efficiency is largely enhanced (|Δ*E*_S1–T1_| < 1.5 kcal/mol) while shifting significantly the emission color toward the deep red visible region and even the IR region is remarkable. The optimal rISC condition (S_1_–T_1_ energy degeneracy) can be even matched for NO_2_-A5, although in this case only a modest shift of the emission color is observed, reaching the green region. All in all, we can conclude that, properly selecting the electronic nature and position of the substituents to be introduced, we can *ad hoc* modulate the TADF process of PTZ-DBTO2, within its physical limits.

## 4 Conclusions

In this work, we have introduced a novel theoretical formalism, by which the energy modulation between two electronic states of a chemically substituted chromophore can be divided in two terms: a term taking into account the structural deformation of the chromophore induced by substitution, called geometrical effect, and a term considering the effect of the substituent group and its interaction with the chromophore—thus including the substituent-induced electronic effect—called differential effect. Within this general framework, the developed formalism was applied to a paradigmatic class of compounds (i.e., PTZ-DBTO2 derivatives) known for their application as OLEDs through TADF. The relevant triplet (T_1_) and singlet (S_1_) electronic states of a wide range of substituents were calculated by DFT and TD-DFT, respectively, including all possible substituent positions and electronic nature. Later, the rationalization of the obtained results made possible to quantitatively clarify the observed trends behind T_1_ → S_1_ rISC and S_1_ → S_0_ emission, in terms of energy, CT, and (rarely considered) spin density. Within the physicochemical limits found for this specific class of compounds, our results point toward the possibility to control the modulation of the two key properties governing TADF: the energy gap between S_1_ and T_1_ states, determining the efficiency of the rISC, and the energy gap between S_1_ and S_0_ states, determining the emission color of the diode. Especially, while the S_1_–T_1_ energy gap should be minimized to increase the efficiency of the process, the S_1_–S_0_ energy gap needs to match a specific value, depending on the emission color of interest. Interestingly, we found that chemical substitution can induce a different effect on these two key properties: while, as it can be expected, the largest possible S_1_–S_0_ energy gap (i.e., a fluorescence blue-shift) can be obtained if electron donor (acceptor) substituents are covalently linked to the donor (acceptor) moiety of the chromophore, the narrowest possible S_1_–T_1_ energy gap (i.e., the most efficient rISC) can be obtained, on the contrary, if electron donor (acceptor) substituents are covalently linked to the acceptor (donor) moiety of the chromophore.

Hence, our results provide a useful guide for predicting the substitution effect on different aspects of OLEDs and, therefore, our presented methodology could be used to design novel materials based on TADF.

## Supporting Information Available

Benchmark calculations and solvent effect; substituent effect on the S_1_–S_0_ energy gap: theoretical formalism; natural transition orbitals (NTOs) and charge transfer (CT) character at T_1_ and S_1_ minima; chemical structure of all the compounds under study; correlation between the S1 CT character and the geometrical/differential effect on the S_1_–T_1_ energy gap; discussion of the four-state model; spin–orbit coupling values; properties computed at the S1 minimum for the S_1_–S_0_ energy gap; application of the Marcus theory; TD-DFT calculation of S1–S0 vs S_1_–T_1_ energy gap; and Cartesian coordinates (PDF)
