# Supplementary material for: Modulating Efficiency and Color of Thermally Activated Delayed Fluorescence by Rationalizing the Substitution Effect
Source: J Chem Theory Comput. 2024 May 13;20(10):4239–53. doi: 10.1021/acs.jctc.4c00009 (PMC11137832; doi:10.1021/acs.jctc.4c00009)
Supplement: Supplementary file 1 — ct4c00009_si_001.pdf [file ct4c00009_si_001.pdf]

Modulating Efficiency and Color of Thermally Activated Delayed Fluorescence by Rationalizing the Substitution Effect

Alejandro Jodra,[1] Marco Marazzi,[1,2] Luis Manuel Frutos,[1,2*] Cristina García-Iriepa[1,2*]*

[1] Universidad de Alcalá, Departamento de Química Analítica, Química Física e Ingeniería Química, Grupo de Reactividad y Estructura Molecular (RESMOL), Ctra. Madrid-Barcelona, Km 33.600, 28871, Alcalá de Henares, Madrid, Spain

[2] Universidad de Alcalá, Instituto de Investigación Química ''Andrés M. del Río'' (IQAR), Ctra. Madrid-Barcelona, Km 33.600, 28871, Alcalá de Henares, Madrid, Spain

Email address of the corresponding authors

marco.marazzi@uah.es (M.M)

cristina.garciai@uah.es (C.G.-I.)

luisma.frutos@uah.es (L.M.F.)

Content

1. Benchmark calculations and solvent effect
2. Substituent effect on the S1-S0 energy gap: theoretical formalism
3. Natural Transition Orbitals (NTOs) and Charge Transfer (CT) character at T_1 and S_1 minima.
4. Chemical structure of all the compounds under study.
5. Correlation between the S1 CT character and the geometrical/differential effect on the S_1-T_1 energy gap.
6. Discussion of the four-states model.
7. Spin-orbit coupling values.
8. Properties computed at the S_1 minimum for the S_1-S_0 energy gap.
9. Application of the Marcus theory
10. TD-DFT calculation of S_1-S_0 vs. S_1-T_1 energy gap
11. Cartesian coordinates
12. References

1. Benchmark calculations and solvent effect

A benchmark of DFT functionals and basis sets has been performed in order to select the most suitable level of theory for this study. Moreover, the effect of considering an implicit solvent has been evaluated, using the Polarizable Continuum Model (PCM).[1]

Table S1. Benchmark of DFT functionals in gas phase using the SVP basis set. Energies are computed in the corresponding electronic state and shown in eV, relative to the S_0 minimum of the axial conformation.

Functional	Axial conformation			Equatorial conformation		
	S_0 min	T_1 min	S_1 min	S_0 min	T_1 min	S_1 min
M062X	0	3.04	3.90	0.04	2.98	3.04
CAM-B3LYP	0	2.86	3.92	0.03	2.87	3.20
B3LYP	0	2.68	-	0.03	2.50	2.00

Table S2. Benchmark of basis sets in gas phase using the M062X DFT functional. Energies are shown in eV, relative to the S_0 minimum of the axial or equatorial conformation.

Basis set	Axial conformation			Equatorial conformation			State selected for calculation
	S_0 min	T_1 min	S_1 min	S_0 min	T_1 min	S_1 min	
SVP	0	0.47	0.30	0	0.40	0.36	S_0
	3.49	3.06	3.11	3.61	2.95	3.04	T_1
	4.22	3.96	3.90	3.63	3.17	3.01	S_1
TZVP	0	0.47	0.35	0	0.44	0.39	S_0
	3.50	3.03	3.10	3.62	2.89	2.99	T_1
	4.20	3.90	3.84	3.70	3.16	3.03	S_1

Table S3. Implicit solvent effects calculated by linear response PCM (LR-PCM, through Integral Equation Formalism) and state-specific corrected-linear response PCM (cLR-PCM). The values for toluene and dichloromethane (DCM) vs gas phase refer to the M062X/SVP level of theory. Energies are computed as gaps between the minimum energy of each potential energy surface and the S_0 minimum of the axial conformation, hence resulting in an equilibrium approach.

	Axial conformation			Equatorial conformation		
	S_0 min	T_1 min	S_1 min	S_0 min	T_1 min	S_1 min
LR-PCM (toluene)	0	3.01	3.83	0.05	2.86	3.11
cLR-PCM (toluene)	0	2.89	3.79	0.05	2.93	2.77
LR-PCM (DCM)	0	2.97	3.78	0.06	2.64	3.16
cLR-PCM (DCM)	0	2.88	3.75	0.06	3.01	2.80
Gas phase	0	3.06	3.90	0.04	2.98	3.04

For LR-PCM calculations, it can be noted that, with respect to gas phase, the S_0-S_1 energy gap is slightly blue-shifted for the equatorial conformation, while it is slightly red-shifted for the axial conformation, when considering both toluene and DCM solvents. In any case, the S_0-S_1 shift is within |0.12| eV, almost within the error of the method.

Concerning the axial conformation, cLR-PCM calculations confirm qualitatively the results obtained by LR-PCM, by slightly stabilizing T_1 and S_1 minima in both toluene and DCM. A higher effect is found for the equatorial conformation, where cLR-PCM calculations indicate a more pronounced stabilization of the S_1 minimum coupled to a destabilization of the T1 minimum.

2. Substituent effect on the S_1-S_0 energy gap: theoretical formalism

An analogous theoretical development as the one presented in the main text for the S_1-T_1 energy gap can be performed for the S_1-S_0 energy gap. First, we define its variation due to substitution as:

$$\Delta\Delta E_{S1-S0}^{R} = \Delta E_{S1-S0}^{R}(\boldsymbol{q}_0^R) - \Delta E_{S1-S0}^{H}(\boldsymbol{q}_0^H) \quad Eq.S1$$

where $\boldsymbol{q}_0^R$ are the geometrical coordinates of the substituted derivative and $\boldsymbol{q}_0^H$ are the coordinates of the reference molecule. In this case, $\boldsymbol{q}_0^R$ refers to the minimum energy structure in the S_1 state for the substituted system, while $\boldsymbol{q}_0^H$ refers to the equivalent point for the unsubstituted system.

The energy differences are defined as:

$$\Delta E_{S1-S0}^{R}(\boldsymbol{q}_0^R) = E_{S1}^{R}(\boldsymbol{q}_0^R) - E_{S0}^{R}(\boldsymbol{q}_0^R) \quad Eq.S2$$

$$\Delta E_{S1-S0}^{H}(\boldsymbol{q}_0^H) = E_{S1}^{H}(\boldsymbol{q}_0^H) - E_{S0}^{H}(\boldsymbol{q}_0^H) \quad Eq.S3$$

In a similar way as presented in the main text for the T_1 state, the energy change induced by the substituent can be expanded in terms of a function series up to first order. This expansion is made around the equilibrium geometry of the unsubstituted system Chr-H (*i.e.*, $\boldsymbol{q}_0^H \equiv \mathbf{0}$). In our case, this corresponds to the S_1 minimum energy structure:

$$E_{S1}^{R}(\boldsymbol{q} - \boldsymbol{q}_0^H) = E_{S1}^{H}(\boldsymbol{q} - \boldsymbol{q}_0^H) + \alpha_{R(S1)} + (\boldsymbol{q} - \boldsymbol{q}_0^H)^{\mathrm{T}}\boldsymbol{\beta}_{R(S1)} \quad Eq.S4$$

where $\alpha_{R(S1)}$ represents the constant shift (independent of the coordinates) that the -R substitution induces on the S_1 state of the unsubstituted (Chr-H) system. Additionally, the $\boldsymbol{\beta}_{R(T1)}$ term provides the first variation of the energy as a function of the coordinates. Similarly to what is done for the T_1 state (see main text), taking the gradient of the previous equation, we obtain:

$$\nabla E_{S1}^{R}(\boldsymbol{q} - \boldsymbol{q}_0^H) = \nabla E_{S1}^{H}(\boldsymbol{q} - \boldsymbol{q}_0^H) + \boldsymbol{\beta}_{R(S1)} \quad Eq.S5$$

This gradient equals zero when $\boldsymbol{q} = \boldsymbol{q}_0^R$, leading to:

$$\boldsymbol{\beta}_{R(S1)} = -\nabla E_{S1}^{H}(\boldsymbol{q}_0^R - \boldsymbol{q}_0^H) \quad Eq.S6$$

where $\boldsymbol{q}_0^R - \boldsymbol{q}_0^H$ is the vector providing the structural displacement of the system after substitution in the S_1 state. Therefore, $\boldsymbol{\beta}_{R(S1)}$ is the force induced by the substituent as it has been defined

elsewhere.[3] This force is responsible for the change in the structure upon substitution (from $\boldsymbol{q}_0^H$ to $\boldsymbol{q}_0^R$) on S_1.

Analogously to the case of the S_1-T_1 energy gap variation (see main text), it is possible to determine the variation of the S_1-S_0 energy gap due to substitution (-R), giving rise to:

$$\left.\begin{aligned} \Delta E_{S1-S0}^R(\boldsymbol{q}_0^R) = E_{S1}^R(\boldsymbol{q}_0^R) - E_{S0}^R(\boldsymbol{q}_0^R) = \{E_{S1}^H(\boldsymbol{q}_0^R) - E_{S0}^H(\boldsymbol{q}_0^R)\} + \{\alpha_{R(S1)} - \alpha_{R(S0)}\} \\ +(\boldsymbol{q}_0^R)^{\mathrm{T}}\{\boldsymbol{\beta}_{R(S1)} - \boldsymbol{\beta}_{R(S0)}\} \\ \Delta E_{S1-S0}^H(\boldsymbol{q}_0^H) = E_{S1}^H(\boldsymbol{q}_0^H) - E_{S0}^H(\boldsymbol{q}_0^H) \end{aligned}\right\} \quad Eq.S7$$

The variation of the S_1-S_0 energy gap due to substitution can be expressed as:

$$\Delta\Delta E_{S1-S0}^R = \{E_{S1}^H(\boldsymbol{q}_0^R) - E_{S1}^H(\boldsymbol{q}_0^H)\} - \{E_{S0}^H(\boldsymbol{q}_0^R) - E_{S0}^H(\boldsymbol{q}_0^H)\} + \{\alpha_{R(S1)} - \alpha_{R(S0)}\} + (\boldsymbol{q}_0^R)^{\mathrm{T}}\{\boldsymbol{\beta}_{R(S1)} - \boldsymbol{\beta}_{R(S0)}\} \quad Eq.S8$$

The $\alpha_{R(S1)} - \alpha_{R(S0)}$ term provides the differential stabilization of the two states due to the presence of the substituent, while the term $(\boldsymbol{q}_0^R)^{\mathrm{T}}(\boldsymbol{\beta}_{R(S1)} - \boldsymbol{\beta}_{R(S0)})$ provides the change of the differential structural effect within the chromophore due to the force induced by the substituent. In the following, we limit our expansion in the energy difference the to zeroth-order term. Analogously to the S_1-T_1 treatment, it has been assumed that the substituent induced forces are basically the same in both states, and therefore $\boldsymbol{\beta}_{R(S1)} - \boldsymbol{\beta}_{R(S0)} \approx 0$.

Finally, the variation on the S_1-S_0 energy gap due to substitution is:

$$\Delta\Delta E_{S1-S0}^R = \{E_{S1}^H(\boldsymbol{q}_0^R) - E_{S1}^H(\boldsymbol{q}_0^H)\} - \{E_{S0}^H(\boldsymbol{q}_0^R) - E_{S0}^H(\boldsymbol{q}_0^H)\} + \{\alpha_{R(S1)} - \alpha_{R(S0)}\} \quad Eq.S9$$

where the first two terms correspond to the geometrical effect that the substitution induced to the chromophore, while the third term corresponds to the zeroth-order differential effect of the substituent on the electronic energy of both S_1 and S_0 states. For simplicity, this expression can be written as:

$$\Delta\Delta E_{S1-S0}^R = \Delta E_{S1-S0}^G + \Delta E_{S1-S0}^D \quad Eq.S10$$

being $\Delta E_{S1-S0}^G = \{E_{S1}^H(\boldsymbol{q}_0^R) - E_{S1}^H(\boldsymbol{q}_0^H)\} - \{E_{S0}^H(\boldsymbol{q}_0^R) - E_{S0}^H(\boldsymbol{q}_0^H)\}$ the geometrical effect of the substituent, and $\Delta E_{S1-S0}^D = \alpha_{R(S1)} - \alpha_{R(S0)}$ the zeroth-order differential effect of the substituent.

3. Natural Transition Orbitals (NTOs) and Charge Transfer (CT) character at T_1 and S_1 minima

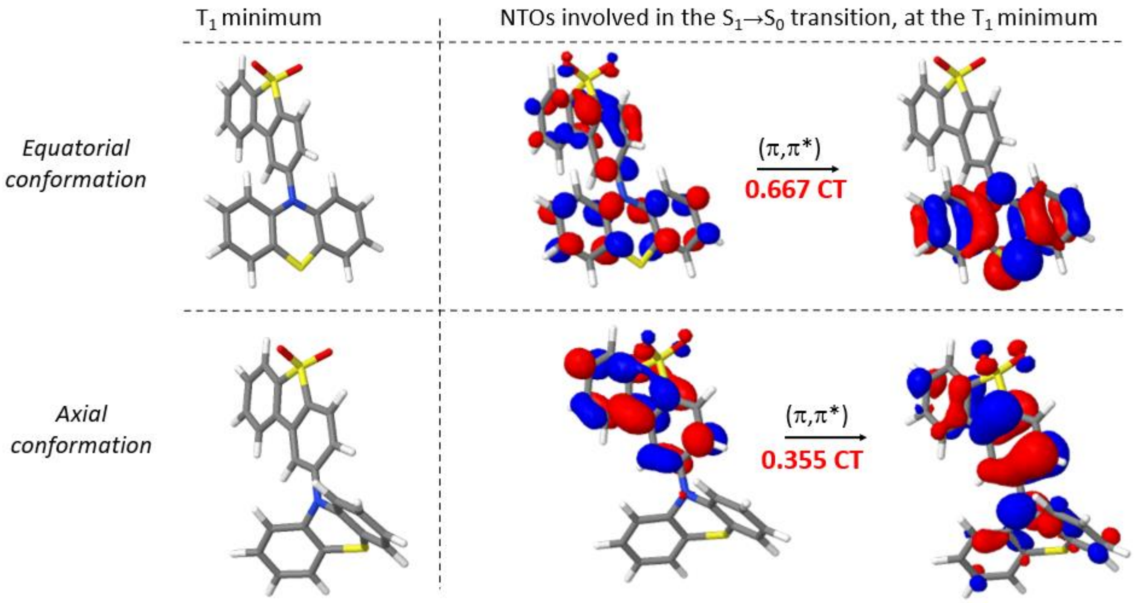

Figure S1. Geometries corresponding to the T_1 minima of the equatorial and axial conformations of the PTZ-DBTO2 reference compound, including the NTOs and the CT character involved in the $S_1 \longrightarrow S_0$ transition.

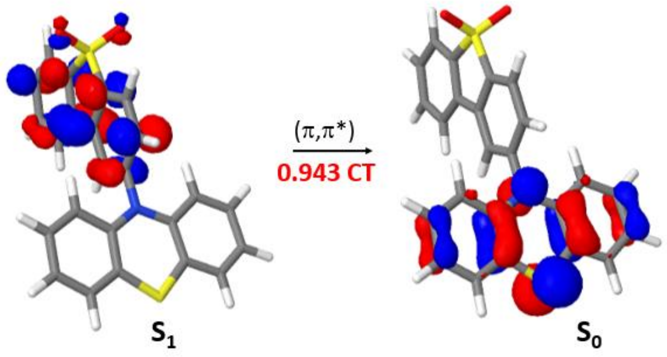

Figure S2. NTOs and CT character involved in the emissive $S_1 \longrightarrow S_0$ transition, at the S_1 minimum of the PTZ-DBTO2 reference compound.

4. Chemical structure of all the compounds under study

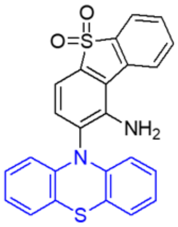

NH₂-A1

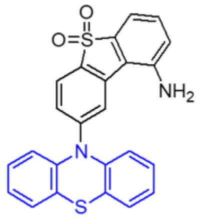

NH_2-A2

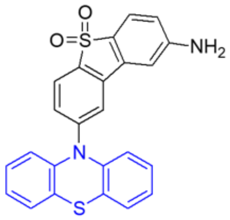

NH_2-A3

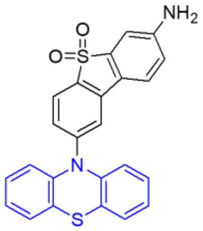

NH_2-A4

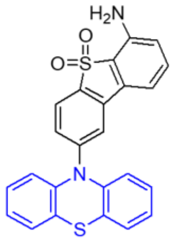

NH_2-A5

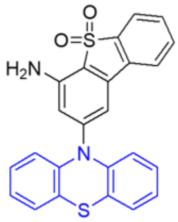

NH_2-A6

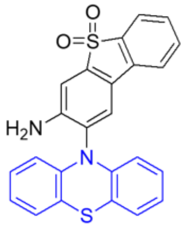

NH_2-A7

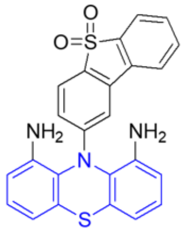

NH_2-D1

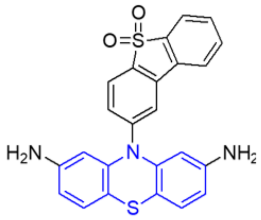

NH_2-D2

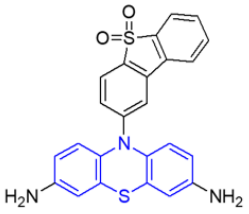

NH_2-D3

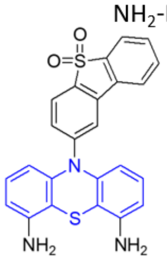

NH_2-D4

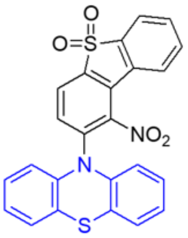

NO_2-A1

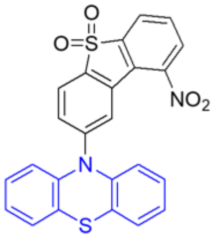

NO_2-A2

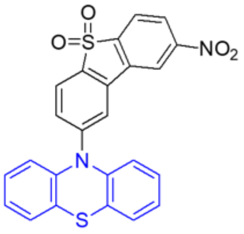

NO_2-A3

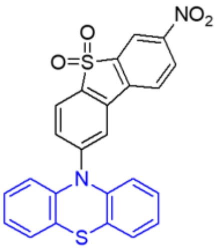

NO_2-A4

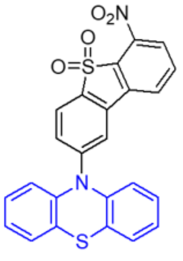

NO_2-A5

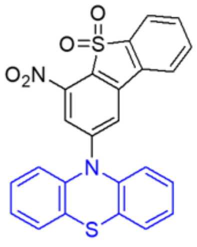

NO_2-A6

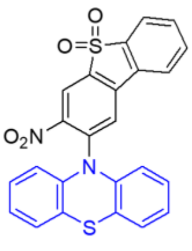

NO_2-A7

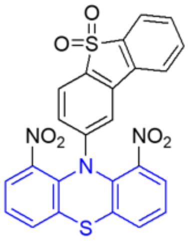

NO_2-D1

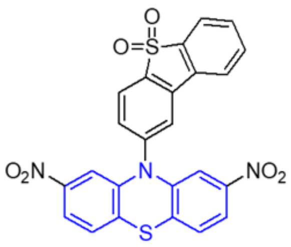

NO_2-D2

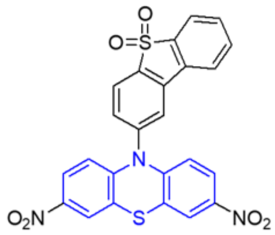

NO_2-D3

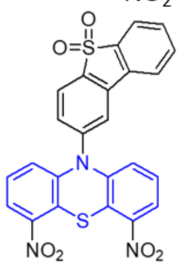

NO_2-D4

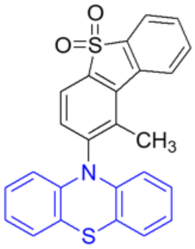

CH_3-A1

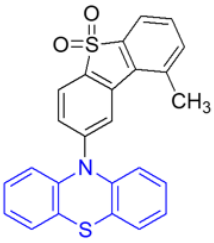

CH_3-A2

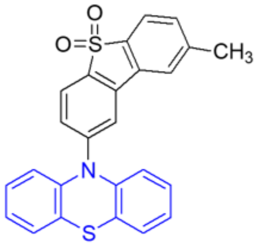

CH_3-A3

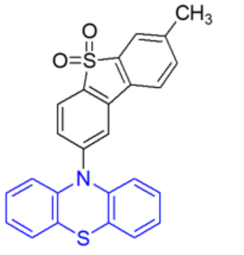

CH_3-A4

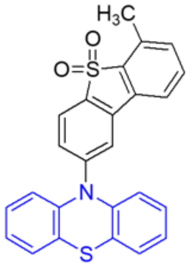

CH_3-A5

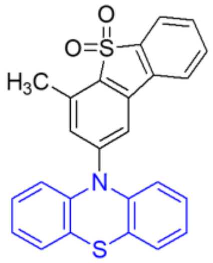

CH_3-A6

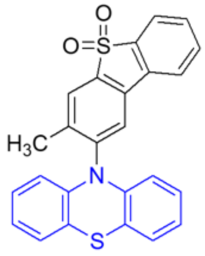

CH_3-A7

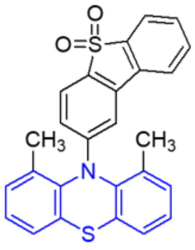

CH_3-D1

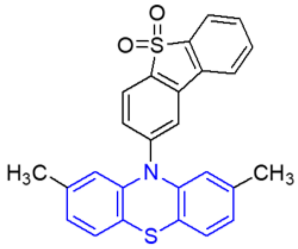

CH_3-D2

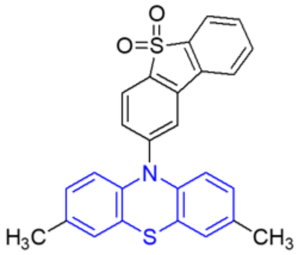

CH_3-D3

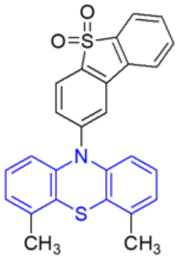

CH_3-D4

Figure S3. Chemical structure of the 33 derivatives under study (reference compound: PTZ-DBTO2).

5. Correlation between the S_1 CT character and the geometrical/differential effect on the S_1-T_1 energy gap

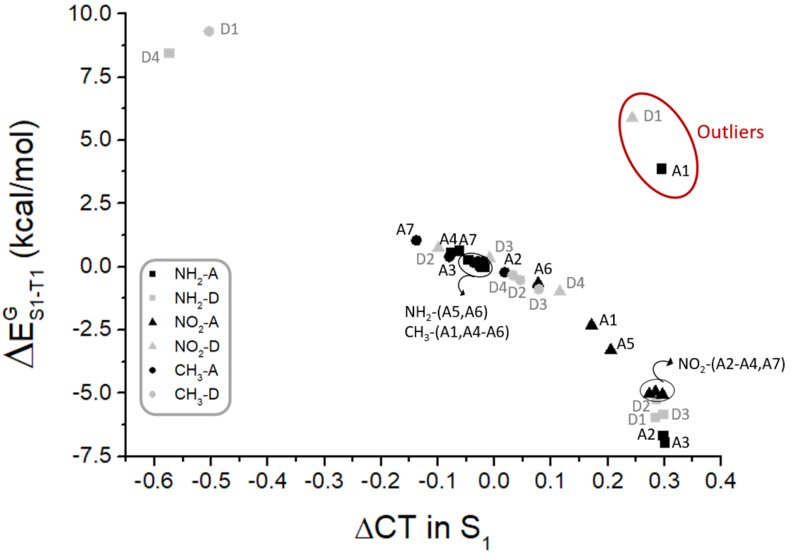

Figure S4. Representation of the geometrical effect on $\Delta\Delta E^{R}_{S1-T1}$ (ΔE^{G}_{S1-T1}) against the variation of the S_1 CT index.

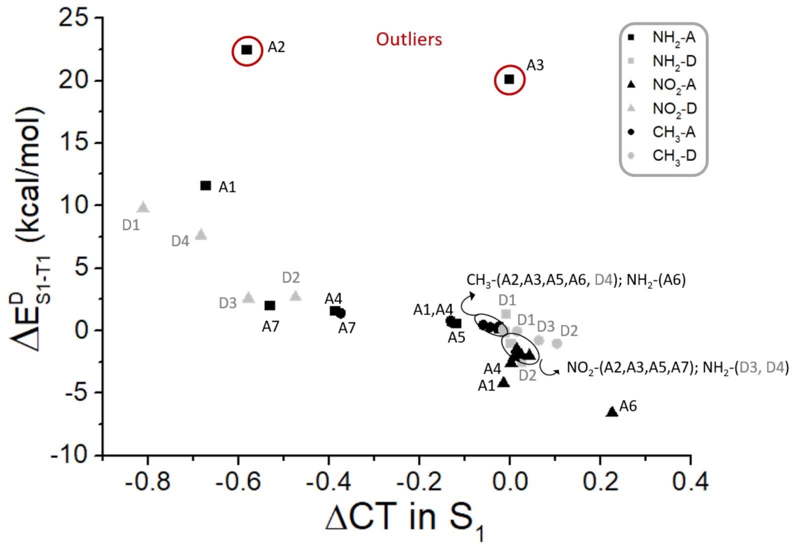

Figure S5. Representation of the differential effect on $\Delta\Delta E^{R}_{S1-T1}$ (ΔE^{D}_{S1-T1}) against the variation of the S_1 CT index.

6. Discussion of the four-states model

In this section we aim to compare our findings – regarding the role of the electronic nature of S_1 and T_1 on the modulation of the S_1-T_1 energy gap, already discussed in the main text – with the published four-states model.[2] For this purpose, the Mulliken charges at the T_1 minimum have been computed for S_0, S_1 and T_1. Then, the charges of the donor and acceptor moieties have been calculated for each electronic state as the sum of the Mulliken charges of the atoms included in each part. Finally, the electronic nature of Locally Excited (LE) or Charge Transfer (CT) states is assigned to S_1 and T_1: an LE state has the same distribution of the charges as in S_0, while for a CT state the charge is localized in a different part of the molecule compared to S_0. Analyzing the results shown in Figure S6, a similar conclusion as the one already discussed in the main text and in agreement with the four-states model[2] can be drawn: both S_1 and T_1 have to be CT states to reach the S_1-T_1 degeneration region.

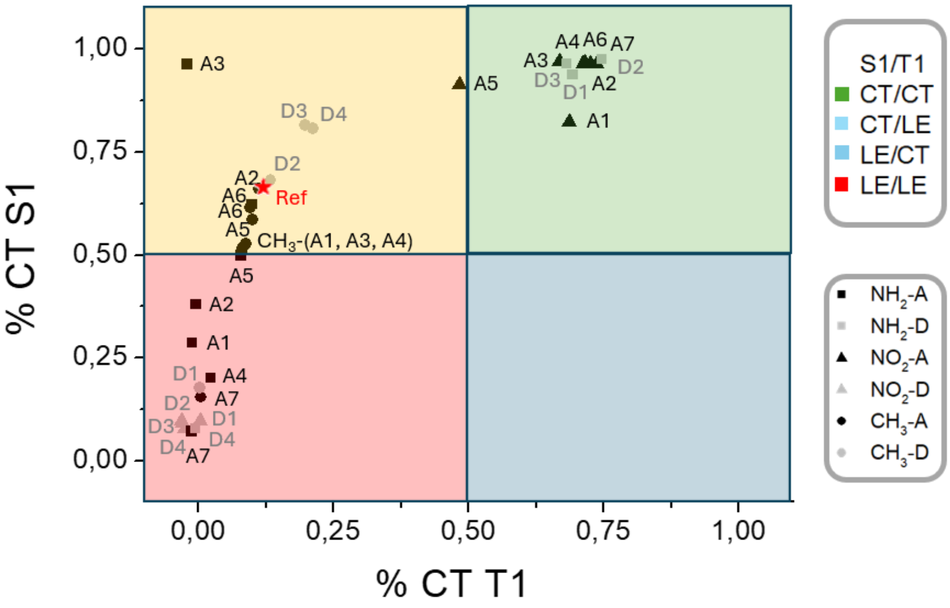

Figure S6. Representation of the S_1 and T_1 CT character, and classification of the compounds regarding the electronic nature of these two electronic states: CT/CT, CT/LE, LE/CT and LE/LE. The reference compound is shown with a red star.

7. Spin-orbit coupling values

Table S4. Sin-Orbit Coupling (SOC) values computed at the T_1 minimum geometries. The SOC value of the reference system is given in the first row. The following rows refer to the derivatives, including for each of them the SOC value and the SOC difference with respect to the reference (ΔSOC).

Substituent	Position	SOC (cm^{-1})	ΔSOC (cm^{-1})
---	----	0.40	0
NH_2	A1	0.28	-0.12
	A2	0.54	0.14
	A3	0.45	0.05
	A4	0.15	-0.25
	A5	0.40	0
	A6	0.44	0.04
	A7	0.09	-0.31
	D1	0.01	-0.39
	D2	0.03	-0.37
	D3	0.06	-0.34
	D4	3.32	2.92
NO_2	A1	0.19	-0.21
	A2	0.02	-0.38
	A3	0.06	-0.34
	A4	0.02	-0.38
	A5	0.18	-0.22
	A6	0.03	-0.37
	A7	0.02	-0.38
	D1	0.17	-0.23
	D2	0.01	-0.39
	D3	0	-0.4
	D4	0.69	0.29
CH_3	A1	0.31	-0.09
	A2	0.43	0.03
	A3	0.34	-0.06
	A4	0.37	-0.03
	A5	0.41	0.01
	A6	0.45	0.05
	A7	0.04	-0.36
	D1	3.04	2.64
	D2	0.42	0.02
	D3	0.42	0.02
	D4	0.38	-0.02

8. Properties computed at the S_1 minimum for the S_1-S_0 energy gap

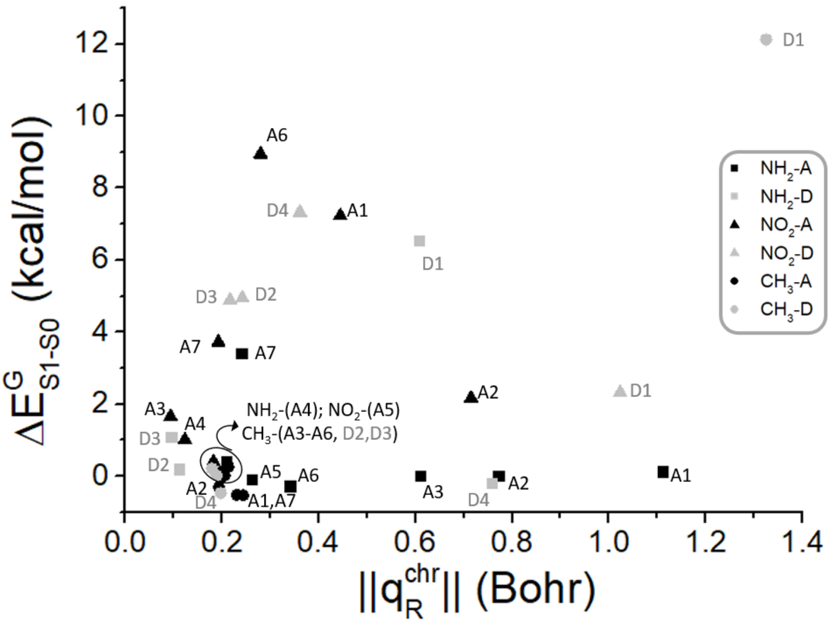

Figure S7. Representation of the geometrical effect contributing to $\Delta\Delta E^{R}_{S1-S0}$ (ΔE^{G}_{S1-S0}) as a function of the modulus of the chromophore coordinates' displacement ($\|\boldsymbol{q}_{R}^{chr}\| \equiv \|\boldsymbol{q}_{0}^{R} - \boldsymbol{q}_{0}^{H}\|$).

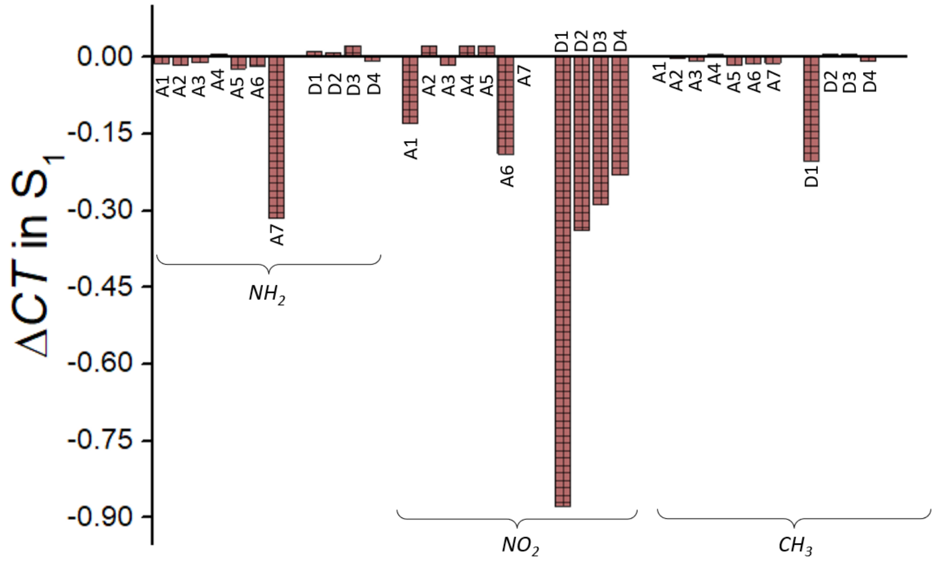

Figure S8. Variation of the CT character in S_1 for the derivatives under study, evaluated at the S_1 minimum, due to the geometrical effect.

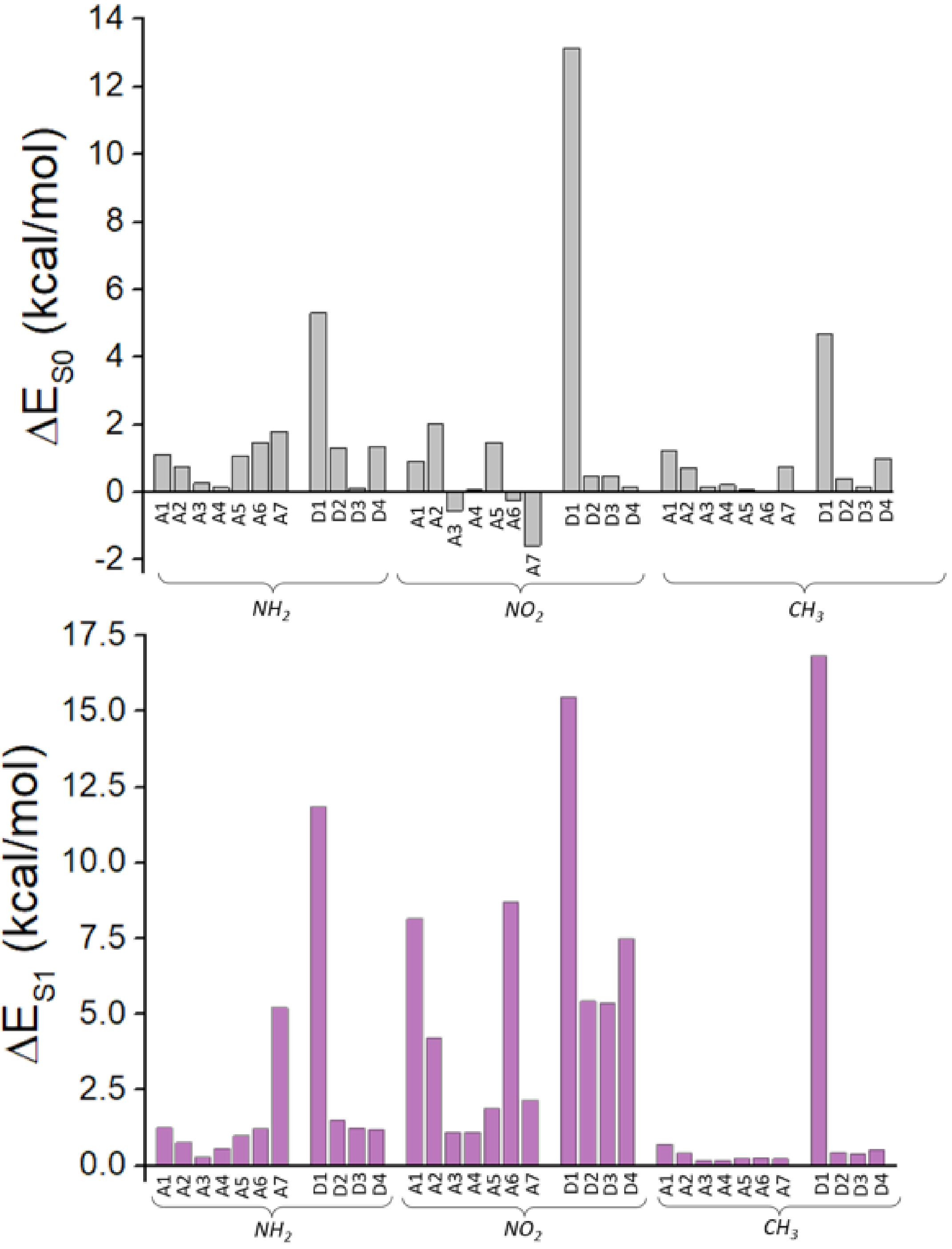

Figure S9. Upper panel: Energy stabilization (negative values) and destabilization (positive values) of S_0 due to the geometrical effect. Lower panel: Energy destabilization of S_1 due to the geometrical effect.

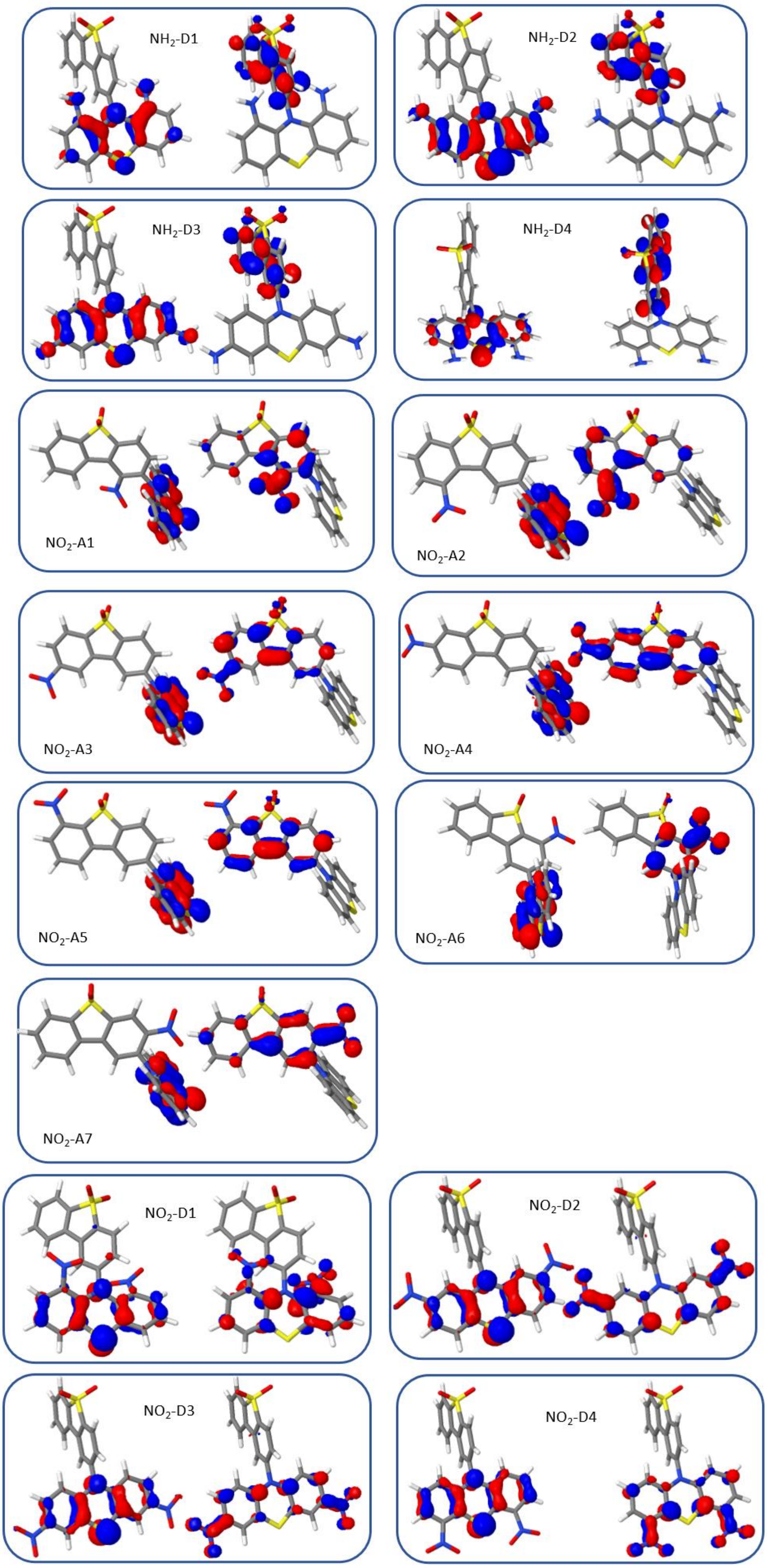

Figure S10. Natural transition orbitals involved in the $S_1 \rightarrow S_0$ transition computed for the S_1 minimum geometry of each compound.

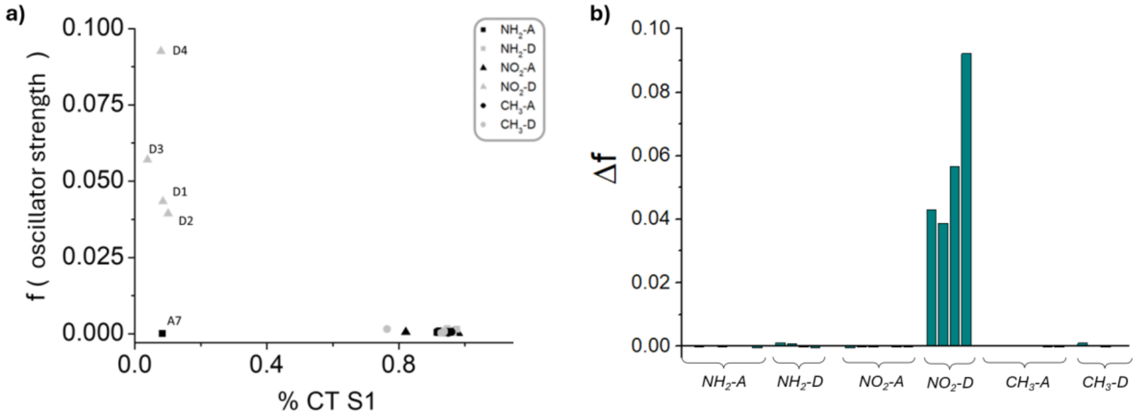

Figure S11. a) S_1-S_0 oscillator strength as a function of the S_1 charge transfer character, and b) variation of the S_1-S_0 oscillator strength with respect to the reference system ($5{\cdot}10^{-4}$), for all the derivatives under study.

9. Application of the Marcus theory

In this work we modulate S_1-T_1 and S_1-S_0 energy gaps through the indicated formalism. In the case of the S_1-T_1 energy gap, such magnitude (ε) can be directly related to the required activation energy ($\Delta G^{\neq}$) to populate the S_1 state from the T_1 minimum.

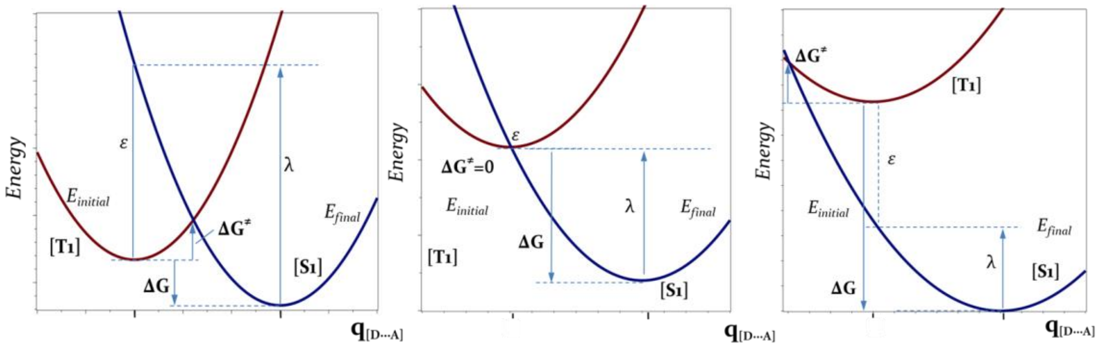

Figure S12. Scheme of the Marcus theory applied to S_1 and T_1 potential energy surfaces, in the case of S_1-T_1 inversion.

Especially, applying the Marcus theory, it can be shown that:

$$\Delta G^{\neq} = \frac{(\lambda + \Delta \mathrm{G})^2}{4\lambda} = \frac{(\varepsilon)^2}{4\lambda} \qquad Eq.S11$$

where λ is the reorganization energy and ΔG is the reaction energy. Since $\Delta G^{\neq}$ depends quadratically from ε, both positive and negative values of ε (left and right panel of Figure S12) result in a positive $\Delta G^{\neq}$, thus lowering the efficiency with respect to $\varepsilon = 0$ (middle panel of Figure S12), corresponding to S_1-T_1 energy degeneracy at the T1 minimum.

10. TD-DFT calculation of S1-S0 vs. S1-T1 energy gap

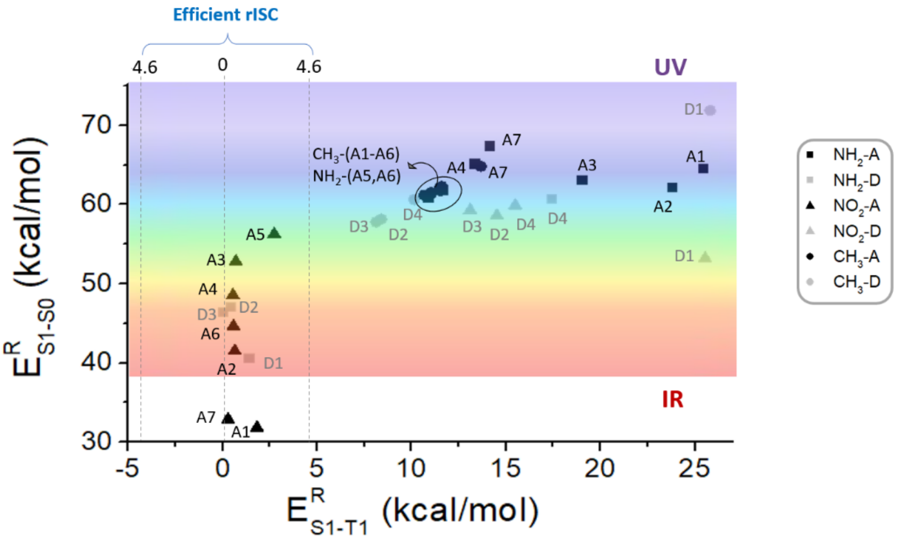

Figure S13. Representation of the S_1-S_0 emission energy gap vs. the corresponding S_1-T_1 energy gap, computed for the 33 derivatives under study, as in Figure 10 of the main text, but using TD-DFT. Slightly higher S_1-T_1 energy gaps are found for all compounds.

11. Cartesian coordinates

S_0 minimum equatorial				T_1 minimum equatorial			
6	0.653183	4.163169	-0.411015	6	0.624977	4.118284	-0.701934
6	2.024180	4.397655	-0.499216	6	1.906157	4.455066	-0.360453
6	2.934734	3.351045	-0.372603	6	2.861493	3.459646	-0.022742
6	2.491840	2.043581	-0.100187	6	2.479982	2.066086	-0.029129
6	1.110344	1.820983	-0.013182	6	1.140456	1.756341	-0.388593
6	0.204460	2.868022	-0.178976	6	0.239884	2.739063	-0.717723
16	4.644025	3.664434	-0.702465	16	4.434620	3.988952	0.379047
6	4.723107	1.220284	0.578794	6	4.709109	1.243703	0.649066
6	5.365295	2.454207	0.367025	6	5.303316	2.558793	0.720146
6	6.628107	2.698870	0.901795	6	6.668088	2.698270	1.088276
6	7.304102	1.709064	1.612933	6	7.439258	1.604328	1.371189
6	6.692956	0.473851	1.795560	6	6.860568	0.296530	1.296514
6	5.413412	0.233213	1.296297	6	5.542050	0.132483	0.950539
7	3.424319	0.992224	0.065526	7	3.378766	1.073569	0.314282
6	2.963275	-1.140664	-1.005608	6	2.952464	-1.027937	-0.832733
6	2.470878	-2.448112	-0.972583	6	2.465368	-2.343117	-0.826580
6	1.939063	-2.906690	0.219465	6	1.928930	-2.826555	0.350194
6	1.875966	-2.123883	1.376224	6	1.846629	-2.074225	1.532859
6	2.368856	-0.823456	1.333061	6	2.333820	-0.763407	1.521117
6	2.911641	-0.338861	0.136433	6	2.875274	-0.271855	0.337688
16	1.250362	-4.537235	0.505961	16	1.250586	-4.468581	0.588547
6	0.878021	-4.139406	2.212422	6	0.859199	-4.118946	2.298702
6	1.264694	-2.834137	2.524300	6	1.233874	-2.815514	2.647767
6	0.284952	-4.990897	3.129386	6	0.264506	-4.999214	3.185753
6	0.071900	-4.503447	4.420951	6	0.033109	-4.551520	4.488981
6	0.450451	-3.201037	4.756060	6	0.398159	-3.254246	4.862209
6	1.047086	-2.358724	3.816235	6	0.996606	-2.381127	3.954179
8	2.293246	-5.544763	0.434630	8	2.302319	-5.466684	0.496272
8	0.025893	-4.708278	-0.255108	8	0.036697	-4.630795	-0.193135
1	-0.051749	4.985961	-0.530986	1	-0.095995	4.893919	-0.959000
1	2.405568	5.402369	-0.690167	1	2.220032	5.501186	-0.342537
1	0.730000	0.817482	0.168921	1	0.820151	0.716124	-0.404793
1	-0.863972	2.657537	-0.116972	1	-0.776879	2.460574	-0.993152
1	7.086726	3.675087	0.734347	1	7.090400	3.704385	1.139162
1	8.300138	1.904218	2.010595	1	8.484976	1.728190	1.651591
1	7.206429	-0.320335	2.338984	1	7.468037	-0.581179	1.515225
1	4.959223	-0.743038	1.455953	1	5.126477	-0.872587	0.905353
1	2.503111	-3.088570	-1.855226	1	2.507233	-2.966982	-1.720597
1	2.342772	-0.171767	2.209184	1	2.301334	-0.123144	2.404162
1	-0.002817	-6.004486	2.846135	1	-0.011267	-6.006767	2.870396
1	-0.392621	-5.143295	5.172021	1	-0.433481	-5.215654	5.217076
1	0.277295	-2.835288	5.768876	1	0.211704	-2.917737	5.882942
1	1.337237	-1.343965	4.093089	1	1.274644	-1.372354	4.263554
1	3.394312	-0.722918	-1.915868	1	3.388369	-0.585366	-1.728686

S_1 minimum equatorial				S_0 minimum axial			
6	0.647139	4.104021	-0.744764	6	5.340622	2.897771	-2.196015
6	1.927058	4.457826	-0.370064	6	5.488726	3.013325	-0.816202
6	2.858745	3.469358	-0.008022	6	4.547376	2.428052	0.033534
6	2.496873	2.095713	-0.010988	6	3.454260	1.734433	-0.500581
6	1.174791	1.762387	-0.403935	6	3.283613	1.671391	-1.885048
6	0.279013	2.745564	-0.763182	6	4.234877	2.234337	-2.731065
16	4.427269	4.031624	0.454979	16	4.705392	2.549893	1.797397
6	4.707239	1.280215	0.661554	6	2.045601	1.991421	1.420405
6	5.303539	2.567370	0.735851	6	2.979257	2.708387	2.180797
6	6.661319	2.711156	1.070427	6	2.548675	3.543843	3.213445
6	7.437878	1.598630	1.321449	6	1.188466	3.662199	3.486933
6	6.862278	0.316719	1.239900	6	0.253762	2.981318	2.705010
6	5.531601	0.154975	0.921705	6	0.681007	2.164064	1.661574
7	3.371833	1.092679	0.360111	7	2.520156	1.132347	0.388990
6	2.952712	-1.016240	-0.819719	6	3.155370	-1.103328	-0.367055
6	2.458739	-2.329493	-0.788634	6	3.051458	-2.487802	-0.281329
6	1.920827	-2.827318	0.375692	6	2.220841	-3.036946	0.680576
6	1.831166	-2.062389	1.598011	6	1.512587	-2.232270	1.573783
6	2.326081	-0.749368	1.563570	6	1.616606	-0.849582	1.495299
6	2.863628	-0.262652	0.386945	6	2.427400	-0.261630	0.502581
16	1.253143	-4.452499	0.599442	16	1.911176	-4.767968	0.966698
6	0.859852	-4.115042	2.301393	6	0.809279	-4.397098	2.331854
6	1.231250	-2.799638	2.675814	6	0.698406	-3.019030	2.532184
6	0.263748	-5.011815	3.173219	6	0.132044	-5.328557	3.100073
6	0.015819	-4.603476	4.483318	6	-0.694691	-4.850401	4.119412
6	0.374187	-3.301814	4.883902	6	-0.820893	-3.476795	4.339636
6	0.970427	-2.408814	4.007058	6	-0.129251	-2.553999	3.552359
8	2.290648	-5.475411	0.488650	8	3.106935	-5.439849	1.447618
8	0.042385	-4.645917	-0.195351	8	1.163284	-5.348511	-0.136653
1	-0.070884	4.874504	-1.025087	1	6.082348	3.346512	-2.857698
1	2.233083	5.505271	-0.350530	1	6.329060	3.563246	-0.389859
1	0.881820	0.715972	-0.421976	1	2.408492	1.155500	-2.283444
1	-0.727860	2.458597	-1.065862	1	4.106530	2.165446	-3.811761
1	7.088575	3.713874	1.126851	1	3.284666	4.105089	3.791267
1	8.490153	1.715976	1.579773	1	0.855523	4.309174	4.299330
1	7.471906	-0.566643	1.429071	1	-0.813076	3.097193	2.899526
1	5.096028	-0.838828	0.860274	1	-0.035669	1.635262	1.030969
1	2.499149	-2.956697	-1.682056	1	3.821150	-0.671185	-1.110746
1	2.291479	-0.111924	2.450314	1	3.621533	-3.125125	-0.959132
1	-0.001492	-6.013764	2.829985	1	1.076362	-0.228090	2.206261
1	-0.451755	-5.285551	5.193038	1	0.244759	-6.397032	2.909618
1	0.177850	-2.987415	5.910507	1	-1.243721	-5.553869	4.746445
1	1.236781	-1.406247	4.347381	1	-1.470398	-3.118389	5.139423
1	3.385978	-0.583475	-1.719015	1	-0.237901	-1.483746	3.735591

T_1 minimum axial				S_1 minimum axial			
6	5.350120	2.881136	-2.179796	6	5.331441	2.855624	-2.216522
6	5.480950	3.099626	-0.810372	6	5.463108	3.126842	-0.856024
6	4.576894	2.513722	0.076251	6	4.570741	2.562136	0.054951
6	3.545257	1.699896	-0.413359	6	3.555325	1.703849	-0.400432
6	3.381073	1.535940	-1.791553	6	3.388664	1.486706	-1.776631
6	4.294798	2.111019	-2.670729	6	4.286651	2.051159	-2.675852
16	4.713756	2.781778	1.823921	16	4.726005	2.896028	1.783470
6	2.103402	2.017422	1.471841	6	2.138697	2.016853	1.499159
6	2.977251	2.856896	2.173482	6	3.001278	2.900687	2.165612
6	2.472271	3.751664	3.119414	6	2.488995	3.781706	3.121503
6	1.101378	3.811836	3.352605	6	1.124538	3.799875	3.388547
6	0.226861	3.010216	2.616204	6	0.257653	2.966103	2.677025
6	0.724225	2.124211	1.665847	6	0.759056	2.084774	1.727632
7	2.660209	1.101957	0.529684	7	2.694993	1.108310	0.556757
6	3.209016	-1.134537	-0.308170	6	3.216253	-1.108387	-0.299495
6	2.989909	-2.506533	-0.311006	6	2.996017	-2.476690	-0.286402
6	2.110560	-3.040445	0.604598	6	2.105345	-3.010128	0.632677
6	1.447386	-2.219967	1.643765	6	1.433290	-2.208823	1.632969
6	1.644455	-0.825915	1.604708	6	1.637172	-0.820805	1.593546
6	2.461048	-0.261884	0.636205	6	2.480129	-0.261833	0.622074
16	1.681945	-4.753553	0.777477	16	1.707685	-4.731038	0.797064
6	0.735220	-4.413785	2.236944	6	0.728608	-4.409762	2.224573
6	0.707424	-3.018788	2.543188	6	0.671593	-3.011987	2.531302
6	0.083795	-5.369268	2.999943	6	0.071905	-5.371132	2.986870
6	-0.633547	-4.949780	4.118331	6	-0.667535	-4.957654	4.086392
6	-0.683318	-3.574857	4.449272	6	-0.742666	-3.577886	4.412222
6	-0.035172	-2.622773	3.691197	6	-0.092631	-2.622455	3.658597
8	2.867720	-5.547968	1.069738	8	2.908259	-5.506440	1.095772
8	0.826508	-5.174697	-0.324115	8	0.902504	-5.166893	-0.341013
1	6.062336	3.336231	-2.868805	1	6.031507	3.300592	-2.924183
1	6.278548	3.733515	-0.420264	1	6.250584	3.788339	-0.492554
1	2.543701	0.940889	-2.159262	1	2.556770	0.870343	-2.118622
1	4.174372	1.965968	-3.744616	1	4.161634	1.872690	-3.744114
1	3.158690	4.407618	3.656769	1	3.166598	4.464544	3.635887
1	0.711201	4.508290	4.095651	1	0.730416	4.490453	4.134833
1	-0.849305	3.081472	2.776425	1	-0.816793	3.010218	2.856481
1	0.055260	1.494828	1.077604	1	0.094450	1.436013	1.156824
1	3.971114	-0.704897	-0.951041	1	3.977996	-0.688286	-0.948122
1	3.525708	-3.151984	-1.009337	1	3.538458	-3.134755	-0.967859
1	1.153516	-0.201458	2.347575	1	1.156188	-0.195830	2.340663
1	0.137103	-6.423892	2.723251	1	0.144316	-6.426780	2.718238
1	-1.153830	-5.678627	4.739615	1	-1.188405	-5.690766	4.702094
1	-1.246491	-3.262104	5.330176	1	-1.325104	-3.268269	5.281342
1	-0.081315	-1.569418	3.972747	1	-0.159687	-1.568247	3.934750

12. References

(1) Tomasi, J.; Mennucci, B.; Cammi, R. Quantum Mechanical Continuum Solvation Models. *Chem. Rev.* **2005**, *105* (8), 2999–3094.

(2) de Silva, P.; Kim, C. A.; Zhu, T.; Van Voorhis, T. Extracting Design Principles for Efficient Thermally Activated Delayed Fluorescence (TADF) from a Simple Four-State Model. *Chem. Mater.* **2019**, *31* (17), 6995–7006.

(3) Fernández-González, M. A.; Frutos, L. M. The concept of substituent-induced force in the rationale of substituent effect. *J. Chem. Phys.* **2021**, *154*, 224106.
